# A novel intronic circular RNA circFGFR1^int2^ up-regulates FGFR1 by recruiting transcriptional activators P65/FUS and suppressing miR-4687-5p to promote prostate cancer progression

**DOI:** 10.1186/s12967-023-04718-y

**Published:** 2023-11-22

**Authors:** Ruyue Wang, Jinjing Zhong, Xiuyi Pan, Zhengzheng Su, Yunyi Xu, Mengni Zhang, Xueqin Chen, Ni Chen, Ting Yu, Qiao Zhou

**Affiliations:** Department of Pathology, West China Hospital, Sichuan University, Chengdu, 610041 China

**Keywords:** circRNA, FUS, P65, miRNA, *FGFR1*, Transcription regulation, Prostate cancer

## Abstract

Fibroblast growth factor receptor 1 (FGFR1) is a core component of the FGFs/FGFR pathway that activates multiple signalling pathways, including ERK1/2, PI3K/AKT, PLCγ, and NF-κB. Aberrant expression of *FGFR1* due to gene amplification, chromosome rearrangement, point mutation, and epigenetic deregulations, have been reported in various cancers. FGFR1 overexpression has also been reported in prostate cancer (PCa), but the underlining mechanisms are not clear. Here we report a novel circular RNA, circFGFR1^int2^, derived from intron 2 of *FGFR1* gene, which is overexpressed in PCa and associated with tumor progression. Importantly, we show that circFGFR1^int2^ facilitates FGFR1 transcription by recruiting transcription activators P65/FUS and by interacting with *FGFR1* promoter. Moreover, we show that circFGFR1^int2^ suppresses post-transcriptional inhibitory effects of miR-4687-5p on FGFR1 mRNA. These mechanisms synergistically promote PCa cell growth, migration, and invasion. Overexpression of circFGFR1^int2^ is significantly correlated with higher tumor grade, Gleason score, and PSA level, and is a significant unfavorable prognosticator for CRPC-free survival (CFS) (*RR* = 3.277, 95% confidence interval: 1.192–9.009; *P* = 0.021). These findings unravelled novel mechanisms controlling *FGFR1* gene expression by intronic circRNA and its potential clinicopathological utility as a diagnostic or therapeutic target.

**Graphic Abstract:**

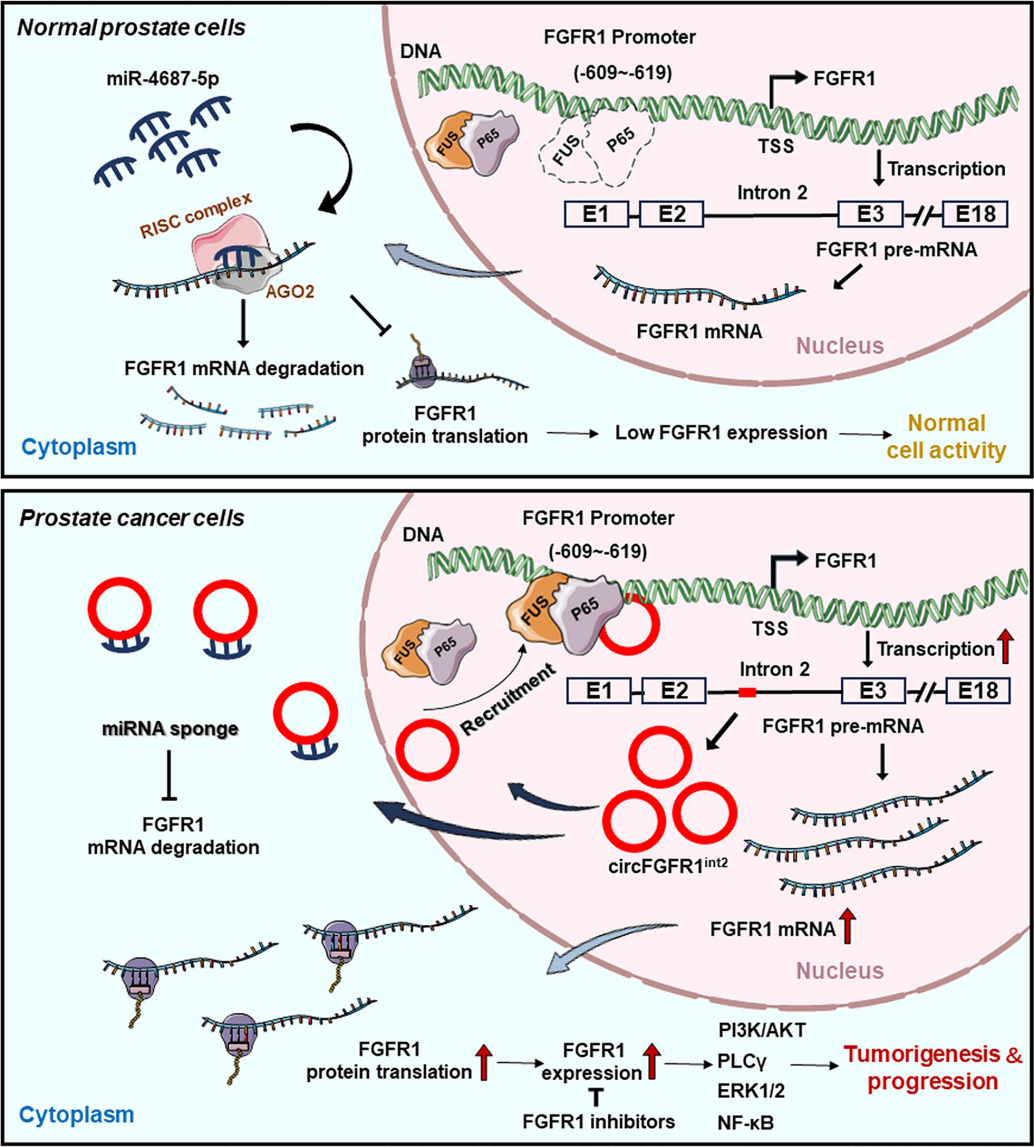

**Supplementary Information:**

The online version contains supplementary material available at 10.1186/s12967-023-04718-y.

## Introduction

Prostate adenocarcinoma (PCa) remains the most prevalent cancer in developed countries and its incidence is rapidly increasing in other countries. Despite significant progresses in diagnosis and treatment, progression from castration sensitive to castration resistant prostate cancer (CRPC) is still a major challenge [1]. Recent studies of PCa tumorigenesis and progression shed lights on many novel molecular abnormalities which may lead to potential new therapeutic approaches, such as *TMPRSS2::ERG* gene fusion, loss-of-function mutations of *SPOP* gene, gain-of-function mutations of *FOXA1*, amplification of *AR* (androgen receptor) and *MYC*, and epigenetic abnormalities such as that mediated by *EZH2* (enhancer of zeste homolog 2) deregulation [2].

The receptor tyrosine kinase fibroblast growth factor receptor 1 (*FGFR1*) has also been reported to be overexpressed in PCa and was associated with PCa progression, angiogenesis, drug resistance, and poorer survival [3]. Interestingly, *FGFR1* has recently been identified as a prospective predictor for advanced PCa by a deep learning model [4].

*FGFR1* is a core component of FGFs/FGFR pathway involved in regulating cell growth, differentiation, and metabolism [5]. FGFR1 is typically activated by fibroblast growth factors (FGFs) [3], leading to activation of signalling pathways such as ERK1/2, PI3K/AKT, PLCγ and NF-κB in PCa [6].

Abnormalities of *FGFR1*, including amplification [7, 8], chromosome rearrangement [9] or gene fusion [10, 11], point mutation [12], and epigenetic deregulation [13, 14] have been reported in a variety of carcinomas, such as lung cancer, alveolar rhabdomyosarcoma, lymphoid neoplasms, extra ventricular neurocytoma, glioma, rosette-forming glioneuronal tumor, and head and neck squamous cell carcinoma.

Noncoding RNAs have recently been described to regulate *FGFR1*. For example, disrupted regulation by miR-133a-3p [15], miR15 and miR16 [16] have been reported in PCa. LncRNA MIR210HG was reported to promote *FGFR1* transcription and glioblastoma multiforme progression [17]. *FGFR1* expression was promoted by circ_SNX27 [18] (in hepatocellular carcinoma) and circRAPGEF5 [19] (in papillary thyroid carcinoma), which suppressed miR-637 and miR-198 respectively.

Here we report the discovery of a novel circRNA (designated circFGFR1^int2^) derived from *FGFR1* intron 2, which was found to be overexpressed in PCa and was associated with PCa progression and unfavorable prognosis. Mechanistically, we found that this novel intronic circFGFR1^int2^ promoted *FGFR1* expression by recruiting the transcription activators *P65* (RELA) and *FUS* (fused in sarcoma), and by suppressing miR-4687-5p, which was found to be an inhibitor of *FGFR1*.

## Materials and methods

### Cell lines and tissue samples

Human PCa cell lines PC-3, DU145, LNCaP, and 22Rv1, normal prostate epithelial cell line RWPE-1, and cervical cancer cell line HeLa were obtained from the ATCC. PCa cells were cultured in RPMI-1640 medium (2,230,740, Gibco, Rockville, MD) with 10% FBS (04–001-1ACS, Biological Industries, Israel). RWPE-1 was maintained in 10% FBS PepiCM (27,774, ScienCell). HeLa cell was cultured in DMEM (11,966–025, Gibco) with 10% FBS.

Formalin-fixed, paraffin-embedded tissue samples of prostate adenocarcinomas (*n* = 62) and benign prostatic hyperplasia (BPH, *n* = 40) diagnosed at the authors’ institution from 2013 to 2018 were used for clinicopathological, immunohistochemistry, in situ hybridization, and survival analysis. All PCa samples were graded according to the 5th edition of WHO classification as described[2], and the grade groups were as follows: Grade Group 1 (*n* = 1, Gleason score ≤ 6, 1.6%), Grade Group 2 (*n* = 12, Gleason score 3 + 4, 19.4%), Grade Group 3 (*n* = 16, Gleason score 4 + 3, 25.8%), Grade Group 4 (*n* = 16, Gleason score 4 + 4/3 + 5/5 + 3, 25.8%), Grade Group 5 (*n* = 17, Gleason score 4 + 5/5 + 4/5 + 5, 27.4%). Fresh tissue samples (PCa, *n* = 6; BPH, *n* = 5) were used for RT-PCR and qRT-PCR analysis. All samples were used according to the institutional ethical guidelines and procedures (including informed consent). Patient disease specific survival (DSS) was defined as the time from PCa diagnosis to death from the disease. Castration-free survival (CFS) was defined as the time from diagnosis to the development of CRPC.

### Antisense oligonucleotides (ASO) and siRNA transfections

Small interfering RNAs (siRNAs) targeting human FUS (si-FUS) and P65 (si-P65), antisense oligonucleotides (ASO) targeting circFGFR1^int2^ (ASO-circFGFR1^int2^), and negative controls were synthesized by RiboBio (Guangzhou, China). The siRNA and ASO sequences were listed in Additional file 1: Table S1. Triplicate siRNA and ASO experiments were performed in both PC-3 and DU145 cells. When cultured PC-3 and DU145 cells reached a density of 70%, the cells were transfected with 100 nM siRNAs or ASO by using the Lipofectamine™ 3000 kit (L3000-015, Invitrogen). The cells were collected for subsequent analysis and experiments 48 h after transfection.

### Nuclear and cytoplasmic RNA fractionation

RNAs were extracted and fractionation by using Invitrogen™ PARIS™ Kit (AM1921; Ambion, Carlsbad, CA). The fractionated RNAs were subjected to PCR analysis, with SNORA41 as nuclear fraction control and GAPDH as cytoplasmic fraction control, respectively.

### Reverse transcription PCR (RT-PCR), quantitative real-time PCR (qRT-PCR), and stem-loop PCR

Total RNA was extracted from cells or tissues by RNAiso Plus (9108, TaKaRa, Dalian, China). HiScript^®^II 1st Strand cDNA Synthesis Kit (R212, Vazyme, Nanjing, China) was used for reverse transcription. RNase R (RNR07250, Epicentre, USA) was used to digest linear RNAs before circRNA analysis. PCR primers were designed, and synthesized by TaKaRa (Additional file 1: Table S2). For miRNA analysis, miRNA-specific primers were used instead of random primers when performing reverse transcription.

RT-PCR was conducted by TaKaRa Taq™ Hot Start Version (R007A, TaKaRa, Dalian, China), PrimeSTAR^®^ HS (R010A, TaKaRa), or 2 × Taq MasterMix (CW0682L, CWBIO, Taizhou, China). Analysis of miRNA was conducted by stem-loop PCR. qRT-PCR was carried out by using the SYBR Green PCR Kit (RR420Q; TaKaRa) and results were analyzed by the 2^−ΔΔCt^ method.

### Western blot analysis

The primary antibodies used were: P65 (rabbit monoclonal, 1:1000, #8242, Cell Signaling Technology); FUS (rabbit monoclonal, 1:1000, #67,840, Cell Signaling Technology); FGFR1 (mouse monoclonal, 1:1000, 60,325-1, Proteintech, Wuhan, China); AGO2 (rat monoclonal, 1:1000, MABE253, Millipore); GAPDH (mouse monoclonal, 1:2000, AG019, Beyotime, Shanghai, China). The secondary antibodies used were: goat anti mouse IgG HRP (1:5000, BS12478, Bioworld); goat anti rabbit IgG HRP (1:5000, BS13278, Bioworld); goat anti rat IgG HRP (1:5000, CW0104, CWBIO). The Immobilon^®^ Western Chemiluminescent HRP Substrate kit (P90719, Millipore) iBright CL1000 (Thermo Fisher Scientific) were used for Western blot imaging.

### In vitro transcription and dot blot

PCR primers were designed, and synthetized by Sango (Shanghai, China) to amplify DNA templates (Additional file 1: Table S3). T7 High Yield Transcription Kit (00874172, Invitrogen) was used for in vitro transcription.

3′-tail biotin-labeled miR-4687-5p probe and negative control probe were synthesized by Sangon (Shanghai, China). RNA templates were serially diluted with DEPC water and then fixed onto nylon membranes (06H04908, Millipore) by ultraviolet. Pre-hybridization was carried out with ULTRAhyb buffer (Ambion) at 37 °C for 1 h and the biotin-labeled miR-4687-5p or negative control probe was added to the reactions for hybridization at 45 °C overnight. Membranes were incubated with HRP-conjugated streptavidin (Thermo Fisher Scientific) and then treated with Immobilon^®^ Western Chemiluminescent HRP Substrate kit and captured by iBright CL1000.

### RNA immunoprecipitation (RIP)

PCa cells were treated by RIP lysis buffer (plus PMSF and RNase inhibitor), sonicated, and centrifuged. Antibodies against AGO2, FUS and P65 were used to precipitate RNAs. Non-immune isotype IgG (03–101, Millipore) was used as negative control. RNA–protein complexes were precipitated by Protein A + G agarose/salmon sperm DNA (P2078, Beyotime). Eluted RNA was purified and used for RT-PCR.

### RNA pull down

3′ tail biotin-labeled circFGFR1^int2^ probes and control probes (Additional file 1: Table S1) were synthesized by Invitrogen (Shanghai, China), and used for pull down of RNA–protein complexes by RNA–Protein Pull Down Kit (20164Y, Thermo Fisher Scientific). Elutes were resolved by SDS-PAGE followed by Western blot analysis.

### Chromatin Immunoprecipitation (ChIP)

Chromatins were crosslinked, sonicated, and fragmented by using the ChIP Kit (P2078, Beyotime). Immunoprecipitation was performed at 4 °C overnight with antibodies against FUS and P65. Protein A + G Agarose/Salmon Sperm DNA was used to precipitate DNA/protein complexes. The precipitated chromatin was analyzed by PCR.

### Co-immunoprecipitation (Co-IP)

PC-3 and DU145 cells were treated with NP-40 cell Lysis Buffer (N8032, Solarbio, Beijing, China) and then sonicated. The supernatants were collected and treated with Sepharose beads (P2078, Beyotime). Anti-FUS or anti-P65 (5 μg) were used for immunoprecipitation, with isotype IgG as negative control. Precipitates were resolved by SDS-PAGE followed by Western blot analysis.

### Chromatin isolation by RNA purification (ChIRP)

circFGFR1^int2^ probes and random probes (Additional file 1: Table S1) were designed, and synthesized by Invitrogen (Shanghai, China). ChIRP was performed as described [20]. Briefly, PCa cells were collected and treated with 1% glutaraldehyde and then lysed in solution buffer containing RNase inhibitor and protease inhibitors. DNA was sheared to 100–500 bp by sonication. The supernatants were hybridized with biotin-labelled circFGFR1^int2^ probes in ChIRP buffers at 37 °C. Eluted complexes were analyzed by PCR and WB.

### Dual luciferase reporter gene assays

Dual luciferase reporter gene assay vectors were constructed by inserting the regulatory sequences in the promoter region or the 3′UTR, respectively. Primers used were listed in Additional file 1: Table S4.

Wildtype *FGFR1* promoter sequences were obtained by genomic DNA PCR and inserted into pGL3-Basic (E1751, Promega). *FGFR1* promoter sequence with mutated P65 binding site was prepared by overlap PCR. The *FGFR1* promoter sequence with mutated circFGFR1^int2^ binding site was synthetized by Sango (Shanghai, China). *FGFR1* 3′UTR sequence or the circFGFR1^int2^ sequence containing miR-4687-5p biding site were obtained by PCR and inserted into 3′UTR region of pGL3-Promoter (E1761, Promega, Madison, WI). *FGFR1* CDS sequence containing the miR4687-5p binding site was cloned downstream of the luciferase CDS of the pMIR-report vector (AM5795, Ambion).

Cells were transfected with reporter constructs, together with miRNA-mimic, siRNAs, or ASO-circFGFR1^int2^. pRL-CMV plasmids (Promega) were co-transfected as internal control. Cells were collected and treated by passive lysis buffer (E1960, Promega). The F (firefly luciferase activity) and R (Renilla activity) values were measured by Dual-Luciferase Reporter Assay System (E1960, Promega) in a fluorescent microplate reader (Synergy2, BioTek, Richmond) and relative luciferase activities (the ratio of F/R) were analyzed.

### Immunohistochemistry (IHC)

Paraffin sections were boiled in 1 × EDTA for antigen retrieval, and incubated with FGFR1 antibody (mouse monoclonal, 1:200, 60,325-1, Proteintech) for 2 h at 37 °C, then with secondary antibody (PV-6000D, ZSGBBIO, Beijing, China) for 1 h at 37 °C. DAB (PV-6000D, ZSGBBIO) was used as chromogen, and hematoxylin for counterstaining.

### In situ hybridization (ISH)

Biotin-labeled circFGFR1^int2^ and negative control probes were designed, and synthetized by Invitrogen (Shanghai, China). Hybridization was performed with the Enhanced Sensitive ISH Detection Kit II (MK1032, Boster). Sections were stained by BCIP/NBT Kit (CW0051S, CWBIO), with methyl green for counterstaining (C0115, Beyotime). Blue-purple circFGFR1^int2^ signals were in the nucleus and cytoplasm of cells were evaluated for intensity and extent, which were scored as previously described [2]. The integrated product of staining intensity and extent ≥ 4 was defined as circFGFR1^int2−high^.

### CCK-8 Cell proliferation assays

CCK-8 assays were performed as described [2]. Cell proliferation was measured at 0 h, 24 h, 48 h, 72 h and 96 h respectively on a spectrophotometer (Bio-TEK FL600, Richmond). OD values at 450 nm were recorded after adding CCK-8 solution (PF725, Dojindo, Japan) for 2 h.

### EdU incorporation assays

EdU incorporation experiment was performed with EdU Kit (C10310-1, RIBOBIO) according to the kit manual as previously described [2]. Results were evaluated by the ratio of EdU-positive cells (red) to total cells (blue).

### Transwell migration and invasion assays

Millicell chambers (MCMP24H48, Millipore, USA) were embedded into 24-well culture dishes before cell culture. Matrigel was overlaid on the surface of chambers for invasion assay. Complete culture medium was added to the lower chambers, while PCa cells suspended in 0.2% FBS RPMI-1640 were added to the upper chambers. Formalin was used for cell fixation, and crystal violet was used for cell staining.

### Wound-healing assays

Cells were seeded onto culture dishes and transfected. When cultured cells reached a density of 90%, straight scratches were drawn by a 1000-μL aseptic pipette tip. The scratch “wound-healing” was recorded by taking photos at 0 h and 48 h respectively. Results were assessed by wound closure ratios (width 0 h-width 48 h / width 0 h).

### Statistical analysis

SPSS Statistics 25.0 (SPSS Inc, USA) and GraphPad Prism 8.0.1 (GraphPad Software, USA) were used for statistical analyses and plotting. Mean ± standard deviation (SD) of three independent replicate experiments was used for quantitative data. Differences between groups were assessed with Fisher's exact test or Mann–Whitney *U*-test. Correlation was assessed by Spearman rank order correlation analysis, with *R* representing the correlation coefficient. The Kaplan–Meier method with log-rank test and Cox proportional regression model were used for survival analysis. Differences were considered significant when *P* < 0.05. The level of significance: *P < 0.05, **P < 0.01, ***P < 0.001; ns, not significant.

## Results

### Identification and characterization of circFGFR1^int2^ in PCa cells and tissues

**Fig. 1:**
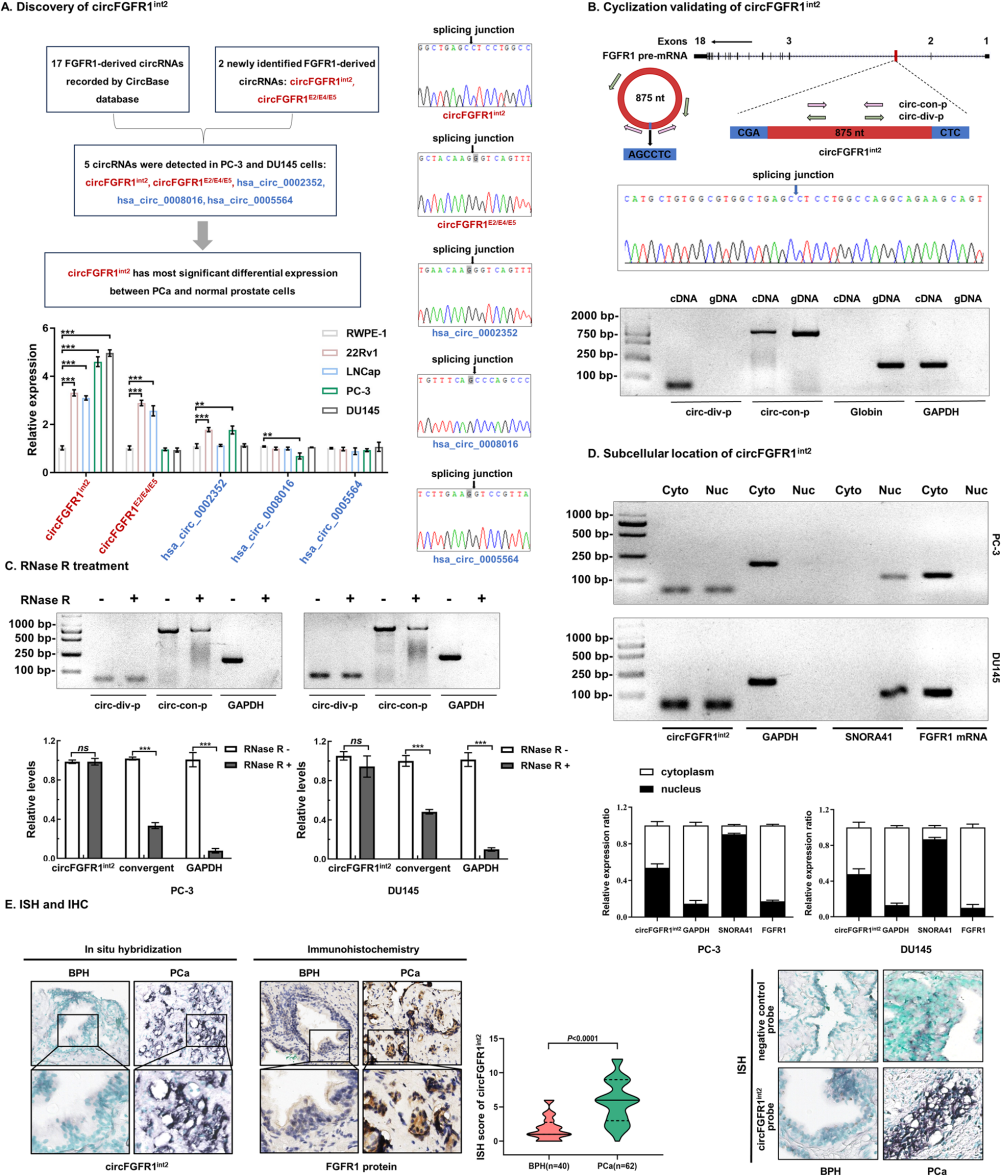
Discovery and characterization of circFGFR1^int2^. **A** Three of the FGFR1-derived circRNAs (hsa_circ_0002352, hsa_circ_0008016, hsa_circ_0005564) recorded in CircBase (*n* = 17) and two newly identified FGFR1-derived circRNAs by the present study (designated circFGFR1^E2/E4/E5^ and circFGFR1^int2^) were expressed in PCa cells. circFGFR1^int2^ was the most abundantly expressed and most significantly differentially expressed between PCa cells (PC-3, DU145, LNCaP, and 22Rv1) and normal prostate epithelium cell (RWPE-1). **B** Further characterization of the novel 875-nt circFGFR1^int2^ by RT-PCR and PCR-sequencing using divergent primers (circ-div-p) spanning splicing junction and convergent primers (circ-con-p). The circFGFR1^int2^ could be amplified from complimentary DNA (cDNA) obtained by reverse transcription, but not from genomic DNA (gDNA). GAPDH and globin were internal controls for PCR. **C** circFGFR1^int2^ was resistant to RNase digestion, as compared to the linear RNA internal control (GAPDH). **D** Cell fractionation showed that circFGFR1^int2^ was distributed in both the cytoplasm (Cyto) and the nucleus (Nuc) fractions, whereas FGFR1 mRNA was located in the cytoplasm. ISH (in situ hybridization) showed strong circFGFR1^int2^ signals (purple) in tumor cell nuclei and cytoplasm of PCa tissues, but only weakly in BPH. **E** Significant higher circFGFR1^int2^ (RNA assessed by ISH) and FGFR1 protein (assessed by IHC) levels in PCa (*n* = 62), but not in BPH (*n* = 40) tissue samples (*P* < 0.0001). Violin plots of ISH data were also shown. Bar charts represented semi-quantitative analysis of RT-PCR experiments (n = 3), with. mean ± standard deviation (SD). *P < 0.05, **P < 0.01, ***P < 0.001

We first performed preliminary analyses of potential novel circular RNAs derived from FGFR1 by RT-PCR and the FGFR1-derived circRNAs recorded in CircBase (http://www.circbase.org/) (Fig. 1A and Additional file 1: Fig. S1) that could be detected in PCa cells, which revealed 5 potential circRNAs expressed in PCa. One of the 5 transcripts showed significantly differential expression in PCa and normal prostate cells, which was a novel transcript not described before. This novel 875nt transcript was generated completely from *FGFR1* intron 2 (Fig. 1A).

PCR with divergent primers (circ-div-p) and convergent primers (circ-con-p) followed by RNase treatment and Sanger sequencing validated the circular nature of this novel transcript, which was designated circFGFR1^int2^ (Fig. 1B and C).

Cell fractionation and tissue in situ hybridization (ISH) experiments showed that circFGFR1^int2^ was distributed in both the nucleus and cytoplasm of PCa cells and tissues (Fig. 1D) in contrast to FGFR1 mRNA, while was primarily enriched in the cytoplasm.

Bioinformatics analyses by ORFfinder, IRESbase, and SRAMP databases revealed several ORFs in the circFGFR1^int2^ sequence, but no IRES (internal ribosome entry sites) or m6A modification sites (Additional file 1: Fig. S2), indicating that circFGFR1^int2^ may not be protein-coding.

### CircFGFR1^int2^ was overexpressed in prostate cancer and promoted FGFR1 expression

**Fig. 2:**
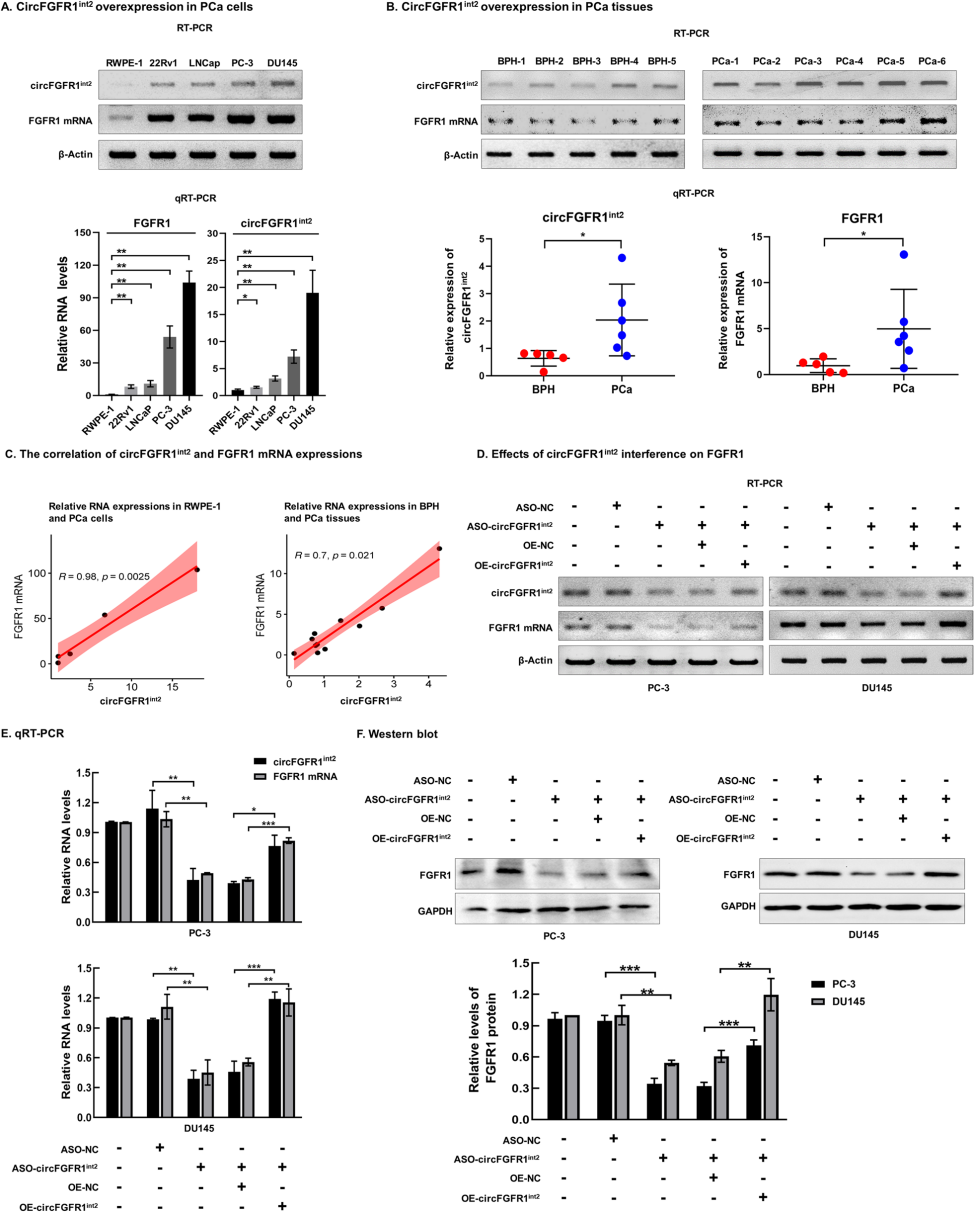
CircFGFR1^int2^ promoted expression of FGFR1 mRNA and protein. **A** CircFGFR1^int2^ and FGFR1 mRNA levels were significantly higher in PC-3, DU145, LNCaP, and 22Rv1 PCa cells than in normal prostate RWPE-1 cells. **B** CircFGFR1^int2^ and FGFR1 mRNA were significantly up-regulated in PCa (*n* = 6) than in BPH (*n* = 5) tissue samples. **C** Expression of circFGFR1^int2^ and FGFR1 mRNA in PCa cells and tissues was significantly correlated. **D**–**F** Knockdown of circFGFR1^int2^ by ASO-circFGFR1^int2^ significantly reduced expression of FGFR1 mRNA (D, RT-PCR; E, qRT-PCR) and protein (**F**, Western blot), whereas artificial overexpression by OE-circFGFR1^int2^ rescued FGFR1 expression. Error bars for RT-PCR, qRT-PCR, and Western blot represented mean ± standard deviation (SD) of three independent experiments. *P < 0.05, **P < 0.01, ***P < 0.001

ISH and immunohistochemistry (ICH) demonstrated that circFGFR1^int2^ and FGFR1 protein were overexpressed in PCa, and the positive rate of circFGFR1^int2^ in PCa was significantly higher than in BPH (*P* < 0.0001) (Fig. 1E). PCR results showed that circFGFR1^int2^ and FGFR1 mRNA were significantly overexpressed in PCa cells 22Rv1, LNCap, PC-3, and DU145, as compared to the normal prostate cell RWPE-1 (Fig. 2A) and BPH tissues (Fig. 2B).

Correlation analysis showed that expression of circFGFR1^int2^ and FGFR1 RNA in prostate cancer were significantly correlated (Fig. 2C). CircFGFR1^int2^ knockdown by antisense oligonucleotides (ASO) significantly decreased the levels of FGFR1 mRNA and protein, while artificial overexpression of circFGFR1^int2^ reversed the effects of ASO (Fig. 2D, E and F). These experiments indicated that circFGFR1^int2^ was a positive regulator of its parental gene *FGFR1*.

### Bioinformatic analysis and experimental validation of circFGFR1^int2^-interacting proteins

**Fig. 3:**
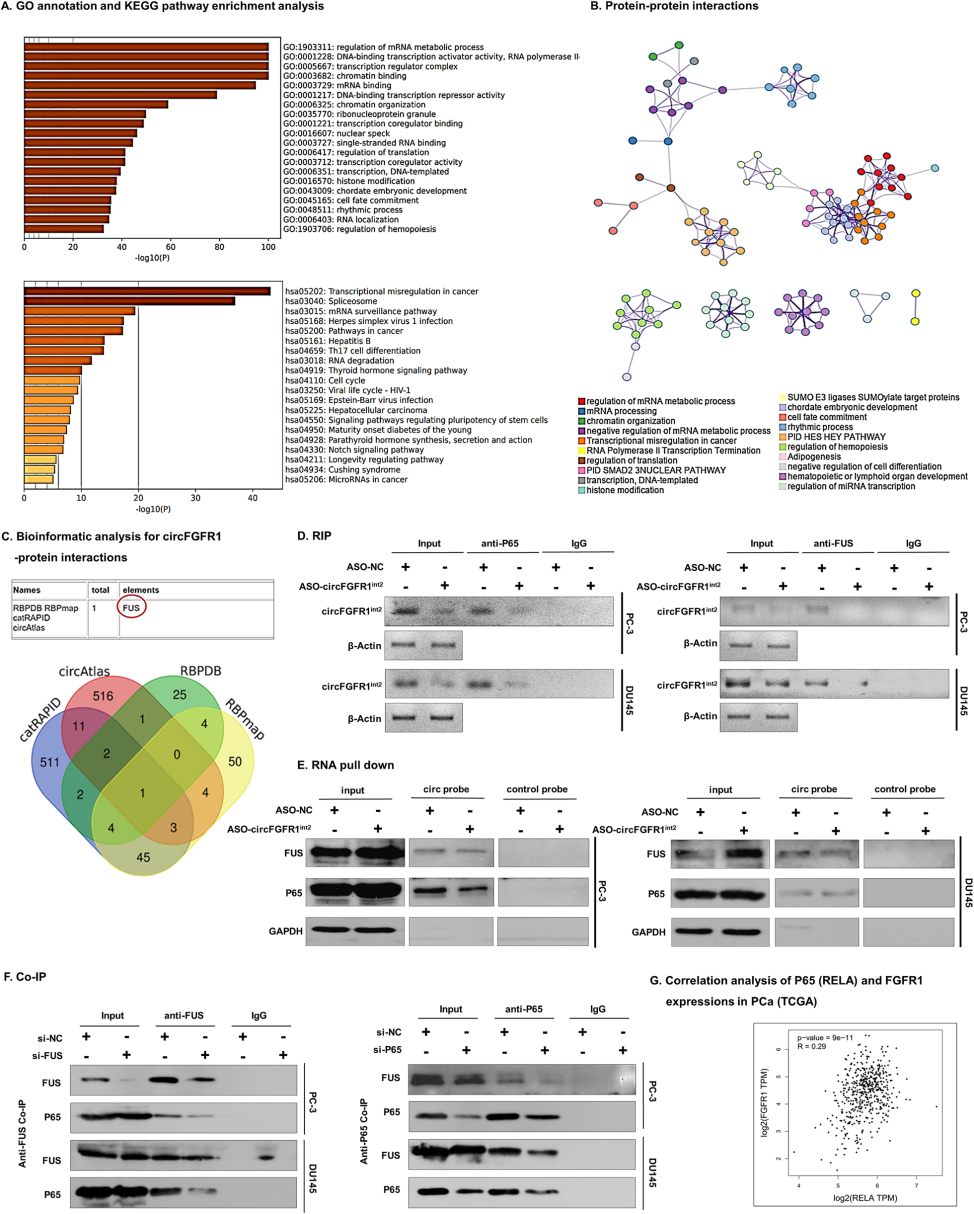
Analysis and identification of circFGFR1^int2^-interacting proteins. **A** Analysis by catRAPID, circAtlas, RBPDB and RBPmap databases revealed potential circFGFR1^int2^-binding proteins and enriched GO terms and KEGG pathways. **B** Analysis by Metascape database (http://metascape.org/gp/index.html#/main/step1) showed potential protein–protein interactions of the circFGFR1^int2^-interacting proteins and the biological processes they may be involved in. **C** The FUS protein was identified as a circFGFR1^int2^-interacting protein by all four databases. **D** RNA immunoprecipitation (RIP) showed retrieval of circFGFR1^int2^ from complexes obtained with both anti-P65 and anti-FUS. Knockdown of circFGFR1^int2^ by ASO-circFGFR1^int2^ decreased recovery of circFGFR1^int2^. Non-immune-IgG was used as negative control. **E** FUS and P65 were pulled down by biotin-labeled circFGFR1^int2^ probe, knockdown of which decreased the recovery. **F** Co-immunoprecipitation (Co-IP) showed retrieval of P65 by anti-FUS, and FUS by anti-P65, respectively. Non-immune-IgG was used as negative control. **G** Expression of P65 and FGFR1 in PCa tissue samples was significantly correlated (TCGA, *n* = 492, *R* = 0.29, *P* < 0.001)

To investigate whether circFGFR1^int2^ functioned as a regulatory RNA by interacting with RNA-binding proteins, bioinformatic analysis via catRAPID, circAtlas, RBPDB and RBPmap databases were performed. A total of 1179 proteins were identified as potential circFGFR1^int2^-interacting proteins by these databases. GO and KEGG pathway analysis (Fig. 3A) and protein–protein interaction analysis by Metascape database (Fig. 3B) showed the potential circFGFR1^int2^-interacting proteins were enriched in mRNA metabolic process, transcription, chromatin binding, mRNA binding, chromatin organization, ribonucleoprotein granule, and histone modification.

Notably, the transcription regulator FUS was identified as a circFGFR1^int2^-interacting protein by all four databases (Fig. 3C). FUS was a co-activator of P65 in the NF-kB signalling pathway [21]. We further tested whether circFGFR1^int2^ interacted with FUS/P65. Cells were first transfected with ASO-circFGFR1^int2^ to reduce endogenous circFGFR1^int2^, and then cell lysates were treated with anti-FUS, anti-P65 or non-immune IgG (negative control). RIP experiments showed that circFGFR1^int2^ and FUS and P65 were co-immunoprecipitated (Fig. 3D). RNA pull down experiments showed that FUS and P65 proteins were recovered by biotin-labeled circFGFR1^int2^ probe, but not by the control probe which consisted of the anti-sense sequence. In addition, when circFGFR1^int2^ was knocked down, the recovery of FUS or P65 was reduced (Fig. 3E). These results indicated that circFGFR1^int2^ co-existed FUS and P65 in a complex.

To test whether FUS and P65 formed a complex, Co-IP experiment was performed, which showed abundant FUS was detected in anti-P65 precipitates, which was decreased by P65 knockdown. Similarly, abundant P65 was detected in anti-FUS precipitates, which was reduced by FUS knockdown (Fig. 3F). These experiments suggested that P65 and FUS coexisted in a complex.

### FUS and P65 promoted FGFR1 transcription

**Fig. 4:**
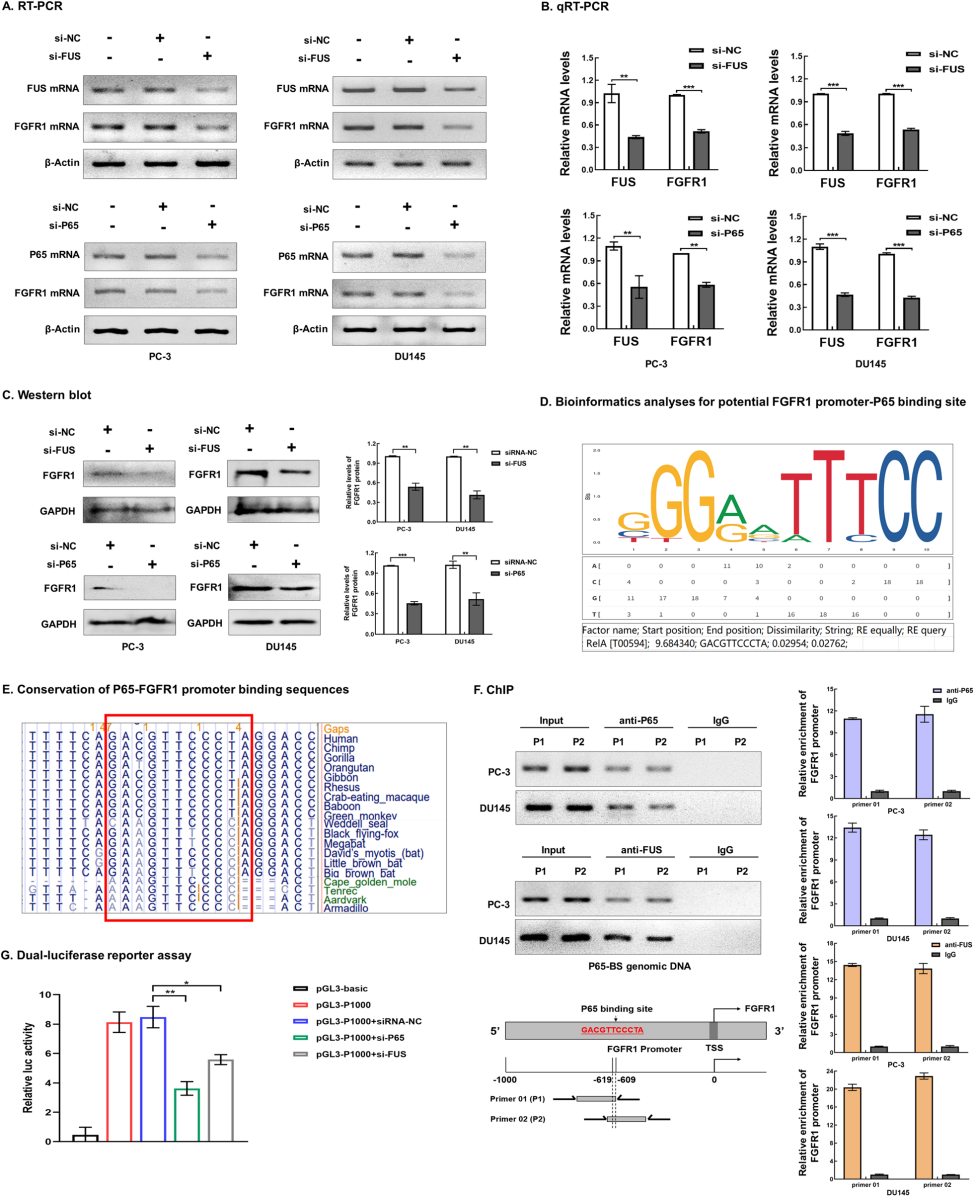
P65/FUS promoted FGFR1 transcription. **A**–**C** Knockdown of P65 or FUS by siRNAs significantly decreased FGFR1 mRNA and protein levels. **D** Predicted P65 binding sequence at -609 ~ -619 upstream of transcription start site by analysis with PROMO (https://alggen.lsi.upc.es/cgi-bin/promo_v3/promo/promoinit.cgi?dirDB=TF_8.3) and JASPAR (https://jaspar.genereg.net/). **E** The predicted P65 binding sequence 5′-GACGTTCCCTA-3′ (in red box) was conserved across species (sequence alignment by UCSC). **F** Chromatin immunoprecipitation (ChIP) with anti-FUS or anti-P65 showed retrieval of FGFR1 promoter sequences containing the P65 binding site (right panels represented qRT-PCR analysis). Input and nonimmune IgG were used for control. **G** Dual-luciferase reporter assays showed that FUS or P65 knockdown by siRNAs significantly decreased the FGFR1 promoter activity. Error bars for qRT-PCR, Western blot, ChIP, and Dual-luciferase reporter assays represented mean ± standard deviation (SD) of three independent experiments. *P < 0.05, **P < 0.01, ***P < 0.001

Expression data from TCGA showed significant positive correlation between P65 and FGFR1 in PCa (Fig. 3G). To test if FUS/P65 promoted FGFR1 expression, knocking down of FUS or P65 by siRNAs was performed, which resulted in significant decrease of the expressions of FGFR1 mRNA (Fig. 4A and B) and protein (Fig. 4C) in PCa cells. Bioinformatics analyses indicated a potential P65-binding site located in the − 609 to − 619 region (DNA sequence: 5′-GACGTTCCCTA-3′) upstream of the transcriptional start site (TSS) of *FGFR1* gene (Fig. 4D). The P65 binding sequence is conserved across species (Fig. 4E). We then performed anti-P65 and anti-FUS ChIP assays to investigate the interactions between *FGFR1* promoter and FUS/P65, which yielded the P65 binding sequence from the chromatin immunoprecipitates obtained by either anti-P65 or anti-FUS (Fig. 4F). These results demonstrated binding of P65 and FUS to the FGFR1 promoter (− 609 to − 619).

To examine whether binding of FUS/P65 to th*e FGFR1* promoter affected *FGFR1* transcription activity, dual-luciferase reporter experiments were performed. The constructs pGL3-P1000 (containing P65 binding site ‘− 609 to − 619’) showed significantly higher relative luciferase activity compared with the baseline activity of pGL3-basic (which lacked the *FGFR1* promoter sequence), either P65 or FUS knockdown by siRNA resulted in significantly lower *FGFR1* promoter activity (Fig. 4G). These experiments indicated that FUS and P65 activated *FGFR1* transcription by binding to the consensus ‘GACGTTCCCTA’ site (Fig. 4G).

### CircFGFR1^int2^ binding to ***FGFR1*** promoter facilitated recruitment of FUS/P65

**Fig. 5:**
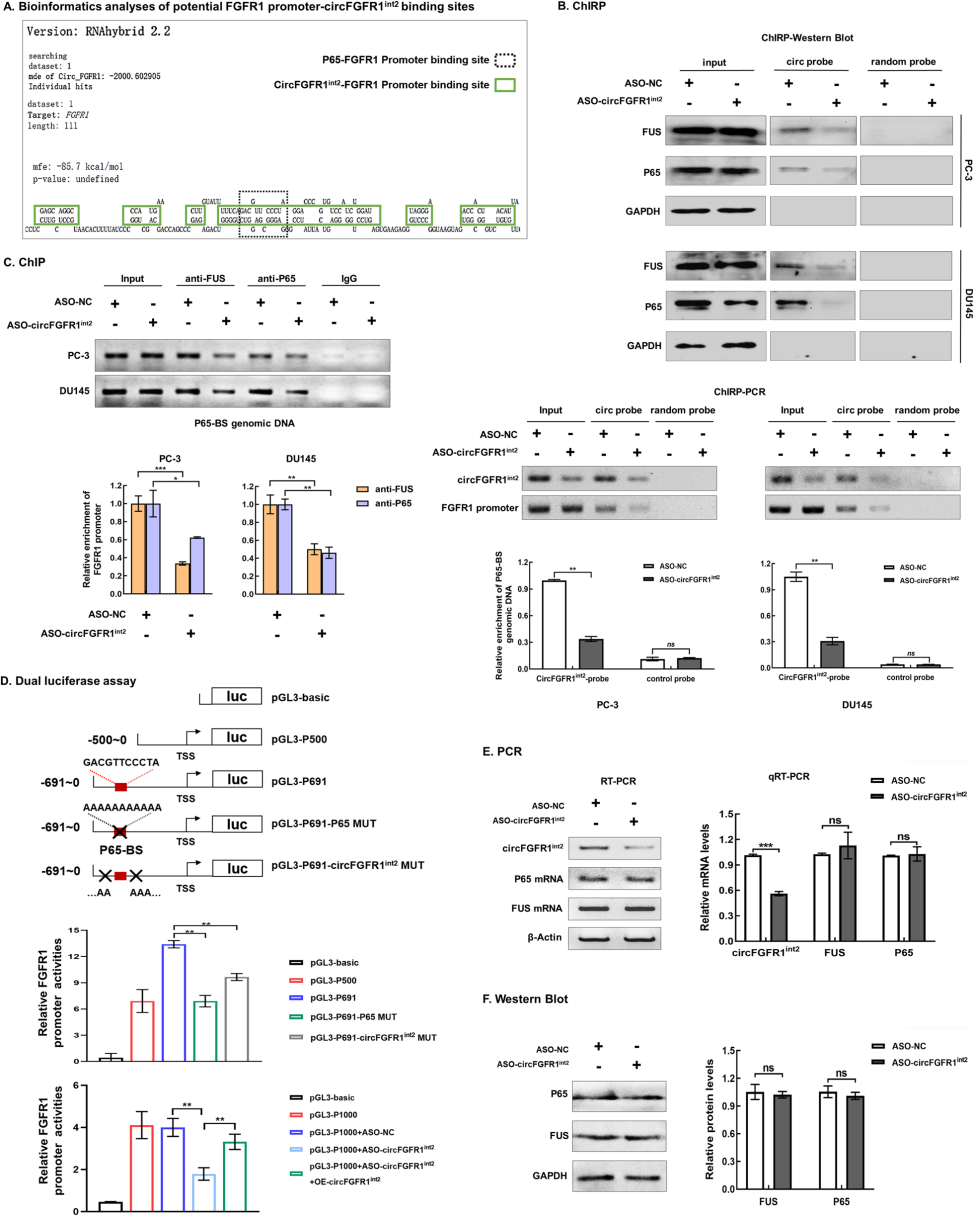
CircFGFR1^int2^ facilitated FGFR1 transcription by recruiting FUS/P65. **A** Bioinformatics analyses by RNAhybrid showed multiple potential circFGFR1^int2^ binding sites (green boxes) enriched around P65 binding site (dotted line box) in the FGFR1 promoter. **B** Chromatin isolation by RNA purification (ChIRP) followed by Western blot analysis and PCR revealed that FUS, P65, circFGFR1^int2^, and FGFR1 promoter sequences containing the P65 binding site were enriched in the complexes obtained with biotin-labelled-circFGFR1^int2^ probes, but not by random-sequence control RNA probe. Knockdown of circFGFR1^int2^ by ASO-circFGFR1^int2^ decreased recovery of the above components. **C** ChIP with anti-FUS or anti-P65 showed that knockdown of circFGFR1^int2^ by ASO-circFGFR1^int2^ significantly reduced retrieval of FGFR1 promoter sequence containing the P65 binding site (lower represented qRT-PCR analysis). **D** Upper: dual-luciferase reporter constructs with wildtype FGFR1 promoter containing P65 binding site (pGL3-P691), or truncated sequence (pGL3-P500) or mutated binding sites (pGL3-P691-P65 MUT, pGL3-P691-circFGFR1^int2^ MUT). Lower: dual-luciferase reporter assays showed significant FGFR1 promoter activity containing P65 binding site (pGL3-P691), which was significantly reduced when the P65 binding site was removed by truncation (pGL3-P500), or when P65 or circFGFR1^int2^ binding sites were mutated (pGL3-P691-P65 MUT and pGL3-P691-circFGFR1^int2^ MUT). Knockdown of circFGFR1^int2^ by ASO-circFGFR1^int2^ significantly decreased the FGFR1 promoter activity, while artificial overexpression of circFGFR1^int2^ by OE-circFGFR1^int2^ rescued the FGFR1 expression. **E**, **F** CircFGFR1^int2^ knockdown by ASO-circFGFR1^int2^ had no significant effects per se on P65 and FUS mRNA and protein expressions. Error bars for ChIRP, ChIP, Dual-luciferase reporter assays, qRT-PCR, and Western blot assays represented mean ± standard deviation (SD) from three independent experiments. *P < 0.05, **P < 0.01, ***P < 0.001, ns, not significant

Bioinformatics analyses by RNAhybrid (http://bibiserv.techfak.uni-bielefeld.de/) indicated multiple potential circFGFR1^int2^ binding sites were enriched in the *FGFR1* promoter, particularly in the 111 bp region surrounding the P65 binding site (− 609 to − 619) (Fig. 5A).

ChIRP experiments were then performed to investigate interactions among P65/FUS/circFGFR1^int2^ and *FGFR1* promoter. Biotin-labelled-circFGFR1^int2^ probes (circ probe) were used for the ChIRP-experiments. Western blot analysis showed that FUS and P65 proteins were enriched by the biotin-labelled-circFGFR1^int2^ probes, whereas knockdown of circFGFR1^int2^ by ASO-circFGFR1^int2^ significantly reduced the retrieval of FUS and P65 proteins (Fig. 5B). These experiments showed that circFGFR1^int2^-FUS/P65 existed in a complex and bound to the *FGFR1* promoter. ChIRP-PCR showed the *FGFR1* promoter sequences containing the P65 binding site (P65-BS genomic DNA) were enriched by biotin-labelled-circFGFR1^int2^ probes, but not by the control probes (random RNA sequences with no complimentary human genomic sequences). CircFGFR1^int2^ knockdown significantly reduced the retrieval of FGFR1 promoter sequences.

To explore whether circFGFR1^int2^ mediated P65/FUS transactivation of *FGFR1*, ChIP assays in which circFGFR1^int2^ was downregulated by ASO-circFGFR1^int2^ were performed, which showed significantly decreased retrieval of FUS or P65 from chromatin immunoprecipitates (Fig. 5C), indicating circFGFR1^int2^ contributed to the binding of P65/FUS to the *FGFR1* promoter.

Luciferase reporter plasmids containing the wildtype (PGL3-P691) or mutated circFGFR1^int2^ binding sites (pGL3-P691-circFGFR1^int2^ MUT), in which all binding sites shown in Fig. 5A were mutated to tandem As were constructed. Dual luciferase reporter assays showed significantly lower *FGFR1* promoter activity in the mutated constructs than wildtype (Fig. 5D). Knockdown of circFGFR1^int2^ by ASO-circFGFR1^int2^ significantly reduced the promoter activity, which was rescued by artificial overexpression of circFGFR1^int2^ (OE-circFGFR1^int2^).

Knockdown of circFGFR1^int2^ by ASO- circFGFR1^int2^ had no significant influence on FUS and P65 mRNA and protein expression (Fig. 5E and F), excluding the possibility that circFGFR1^int2^ functioned by directly affecting FUS and P65 mRNA and protein expression.

### miR-4687-5p was a circFGFR1^int2^-interacting miRNA

**Fig. 6:**
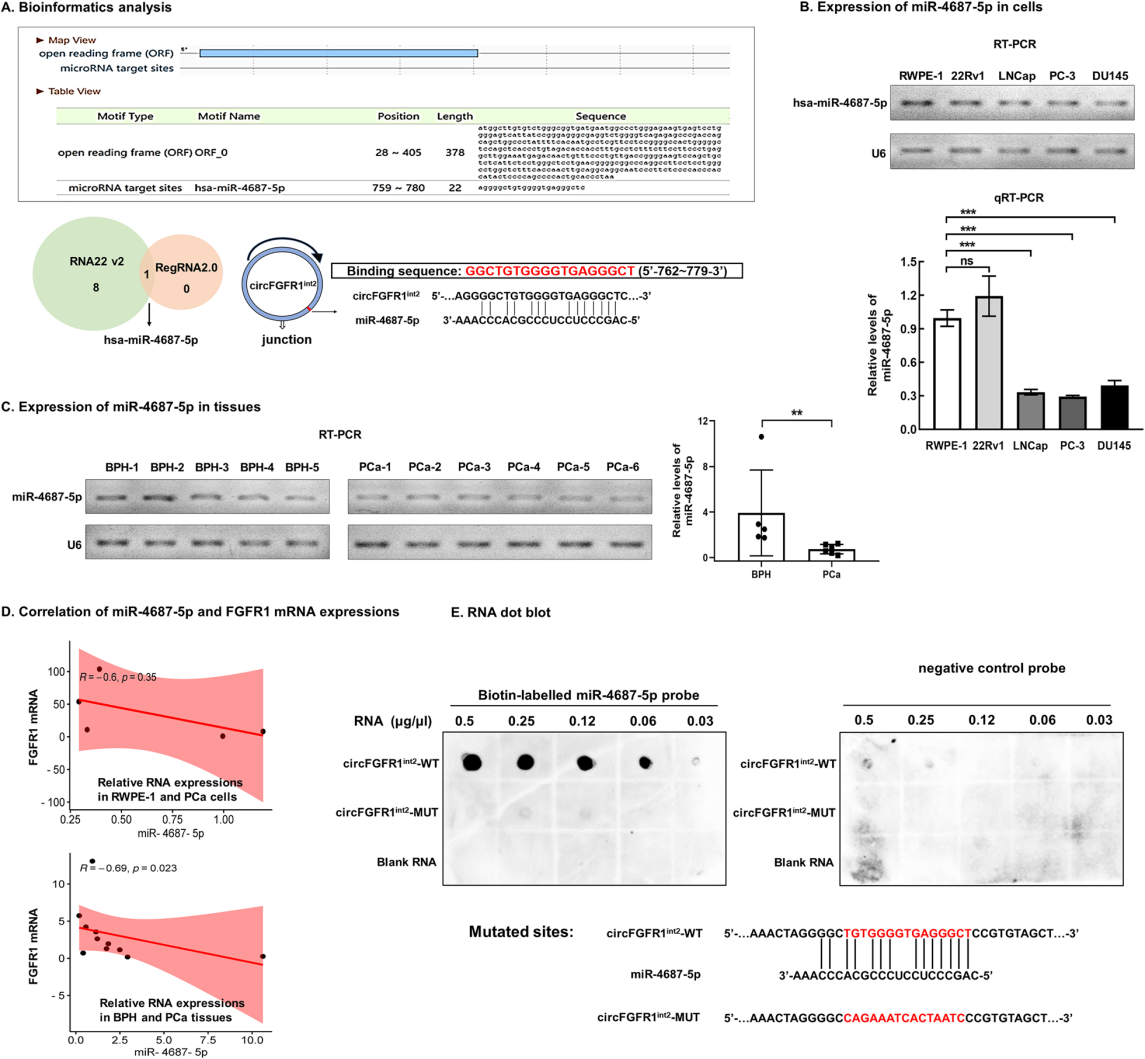
miR-4687-5p was downregulated in PCa and interacted with circFGFR1^int2^. **A** Bioinformatics analysis (RegRNA 2.0 and RNA22 v2) of hsa-miR-4687-5p showing the potential circFGFR1^int2^-interacting sequence TGTGGGGTGAGGGCT (in red) at 762 to 779. **B**, **C** miR-4687-5p was significantly down-regulated in PCa cells (LNCap, PC-3, and DU145) and tissue samples (*n* = 6) than in normal prostate cell RWPE-1 and BPH tissues. **D** Expression of FGFR1 mRNA was negatively correlated with that of miR-4687-5p. **E** Dot blot hybridization showed biotin-labelled miR-4687-5p probe bind to wild-type circFGFR1^int2^ (WT) in dose-dependent manner, whereas mutation of the binding site (MUT) resulted in no or very weak signals. Blank (no RNA was added) was used as negative control. Error bars for qRT-PCR represented mean ± standard deviation (SD) of three independent experiments. **P < 0.01, ***P < 0.001, ns, not significant

Bioinformatics analysis by using RNA22 v2 (https://cm.jefferson.edu/rna22v2/) and RegRNA 2.0 databases (http://regrna2.mbc.nctu.edu.tw/) revealed that hsa-miR-4687-5p was a highly probable target of circFGFR1^int2^, containing a potential 14nt complementary sequence to interact with circFGFR1^int2^ (Fig. 6A).

miR-4687-5p was significantly down-regulated in PCa cells LNCap, PC-3, and DU145 (Fig. 6B) and PCa tissues (Fig. 6C) as compared to normal prostate cells RWPE-1 and 22Rv1 and BPH tissues. Expression of FGFR1 mRNA and miR-4687-5p were negatively correlated (Fig. 6D).

RNA dot blot analysis showed dose-dependent binding of miR-4687-5p to the circFGFR1^int2^ wild type (WT), but not to the mutated circFGFR1^int2^ (MUT) (in which the miR-4687-5p binding site was mutated) (Fig. 6E).

### miR-4687-5p suppressed FGFR1 expression by targeting FGFR1 3′UTR and CDS

**Fig. 7:**
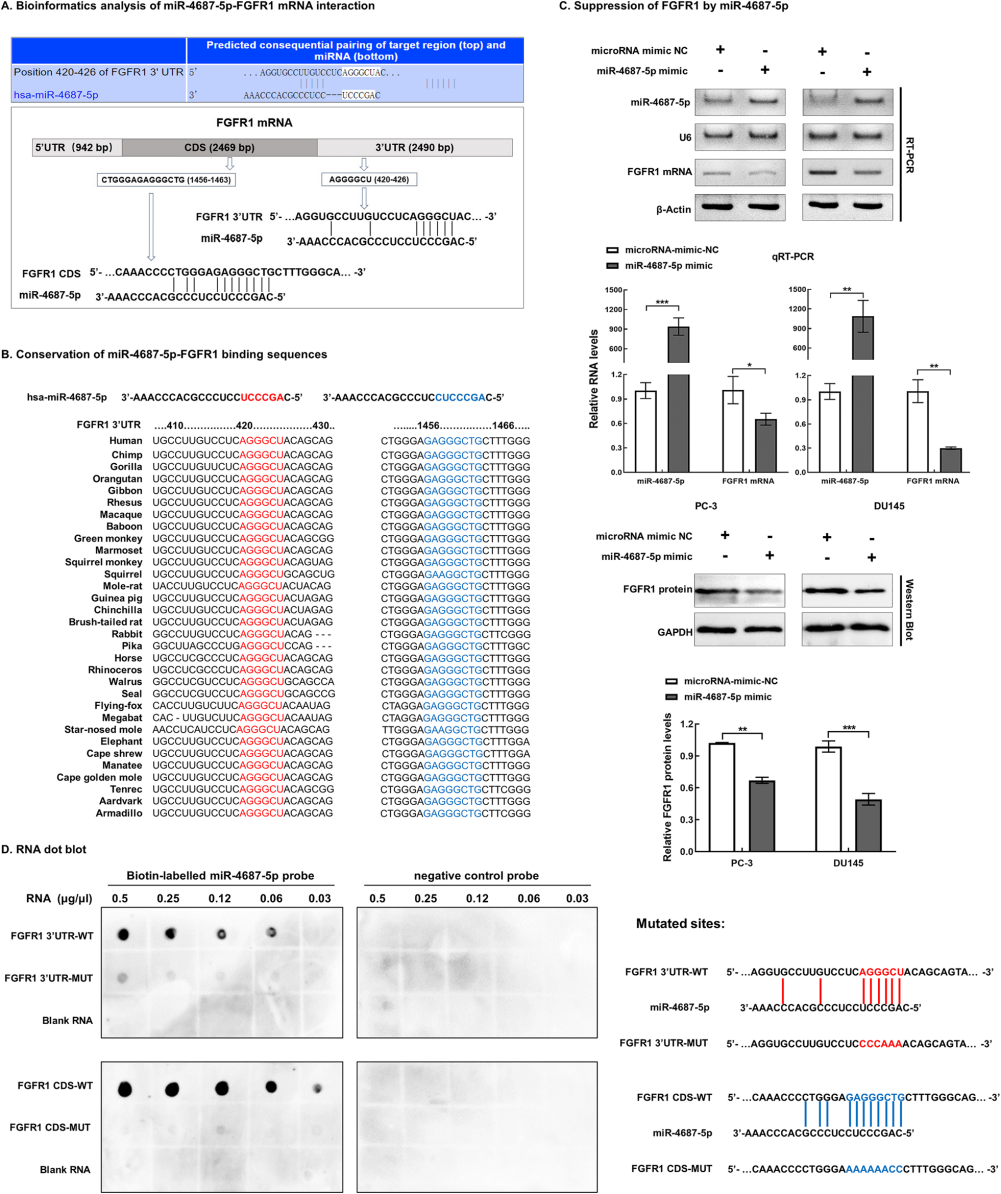
miR-4687-5p was a novel suppressor of FGFR1 by targeting FGFR1 3′UTR and CDS. **A** Bioinformatics analysis by TargetScan, miRwalk, Microt4, miRanda and RNAhybrid databases revealed two potential miR-4687-5p-interacting sites located in FGFR1 3′UTR and CDS, respectively. **B** The miR-4687-5p binding sequences in 3′UTR (5′-AGGGCU-3′, red) and CDS (5′-GAGGGCTG-3′, blue) were highly conserved across species. **C** Artificial overexpression of miR-4687-5p by mimics significantly suppressed expression of FGFR1 mRNA and protein. **D** Dot blot hybridization showing biotin-labelled miR-4687-5p probe bound to wild-type FGFR1 3′UTR and CDS fragments in a dose-dependent manner, while mutation of miR-4687-5p binding sites in FGFR1 mRNA (FGFR1 3′UTR-MUT and FGFR1 CDS-MUT) resulted in no or signals. Error bars for qRT-PCR represented mean ± standard deviation (SD) of three independent experiments. *P < 0.05, **P < 0.01, ***P < 0.001

Bioinformatics analyses by using the miRwalk (http://mirwalk.umm.uni-heidelberg.de/), Microt4 (http://diana.imis.athenainnovation.gr/DianaTools/index.php?r=site/index), TargetScan (https://www.targetscan.org/vert_72/), miRanda (http://www.microrna.org/microrna/home.do) and RNAhybrid (http://bibiserv.techfak.uni-bielefeld.de/) databases showed that there were two potential miR-4687-5p binding sites in FGFR1 mRNA, one in the *FGFR1* 3′UTR (5’-420–426-3′) and the other in the CDS (5’-1456–1463-3′), respectively (Fig. 7A). These response elements (MREs) were highly conserved across species (Fig. 7B).

Artificial overexpression of miR-4687-5p by mimics significantly suppressed the FGFR1 mRNA and protein expression (Fig. 7C). Dot blot analysis showed dose-dependent binding of miR-4687-5p to *FGFR1* 3′UTR-WT and FGFR1 CDS-WT, but not the FGFR1 3′UTR-MUT and *FGFR1* CDS-MUT mRNAs in which the miR-4687-5p binding sites where mutated (Fig. 7D).

### CircFGFR1^int2^ supressed the inhibitory effects of miR-4687-5p on FGFR1

**Fig. 8:**
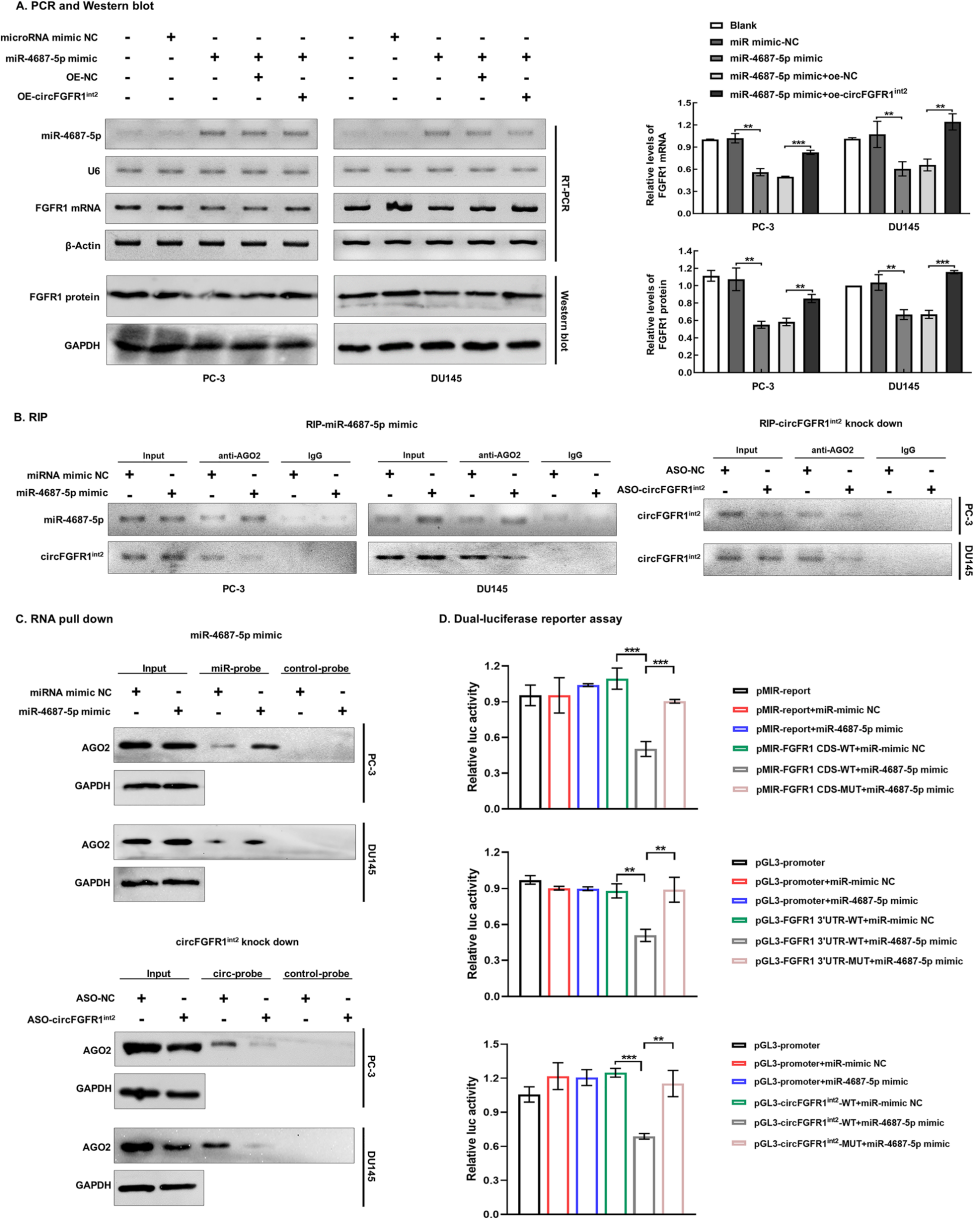
CircFGFR1^int2^ suppressed the inhibitory effects of miR-4687-5p on FGFR1. **A** Artificial overexpression of miR-4687-5p by miR-4687-5p mimic significantly decreased expressions of FGFR1 mRNA and protein in PCa cells, which could be reversed by circFGFR1^int2^ artificial overexpression (OE-circFGFR1^int2^). **B** CircFGFR1^int2^ and miR-4687-5p were simultaneously precipitated by anti-AGO2 in PCa cells. Artificial overexpression of miR-4687-5p or knockdown of circFGFR1^int2^ decreased the recovery of circFGFR1^int2^. **C** Artificial overexpression of miR-4687-5p increased AGO2 pull down by biotin-labeled miR-4687-5p probe, whereas knockdown of circFGFR1^int2^ decreased AGO2 retrieval. **D** Artificial overexpression of miR-4687-5p mimic significantly suppressed the promoter activity of the WT constructs containing the miR-4687-5p binding sited (pGL3-FGFR1 3′UTR-WT, pMIR-FGFR1 CDS-WT, and pGL3-circFGFR1^int2^-WT), which could be rescued by the respective mutant plasmids (pGL3-FGFR1 3′UTR-MUT, pMIR-FGFR1 CDS-MUT and pGL3-circFGFR1^int2^-MUT). Error bars for qRT-PCR and Dual-luciferase reporter assays represented mean ± standard deviation (SD) of three independent experiments. **P < 0.01, ***P < 0.001

Artificial overexpression of miR-4687-5p by mimics significantly decreased the expression of FGFR1 mRNA and protein in PCa cells, which could be reversed by artificial circFGFR1^int2^ overexpression (Fig. 8A), indicating that circFGFR1^int2^ supressed the effects of miR-4687-5p.

CircFGFR1^int2^ and miR-4687-5p were simultaneously precipitated by anti-AGO2 in PCa cells. Artificially overexpression of miR-4687-5p increased the recovery of miR-4687-5p but decreased the recovery of circFGFR1^int2^. Knockdown of circFGFR1^int2^ reduced recovery of circFGFR1^int2^ in AGO2 immunoprecipitation (Fig. 8B). Artificial overexpression of miR-4687-5p increased AGO2 retrieval in pull down by biotin-labeled miR-4687-5p probe, and knockdown of circFGFR1^int2^ decreased AGO2 retrieval from pulldown by biotin-labeled circFGFR1^int2^ probe, suggesting their co-existence in the RISC complex (Fig. 8C).

Dual luciferase assays showed that miR-4687-5p mimic transfection significantly suppressed the relative luciferase activities of the constructs pGL3-circFGFR1^int2^-WT, pGL3-FGFR1 3′UTR-WT and pMIR-FGFR1 CDS-WT (each containing the respective miR-4687-5p binding sites), which could be rescued by pGL3-circFGFR1^int2^-MUT, pGL3-FGFR1 3′UTR-MUT and pMIR-FGFR1 CDS-MUT (each containing the mutated miR-4687-5p binding sites) (Fig. 8D). These experiments showed that miR-4687-5p interacted with *FGFR1* 3′UTR and CDS to inhibit FGFR1 expression, whereas circFGFR1^int2^ suppressed the inhibitory effects of miR-4687-5p on FGFR1 by competitively binding to miR-4687-5p.

### Upregulated circFGFR1^int2^ and downregulated miR-4687-5p promoted PCa cell proliferation, migration, and invasion

**Fig. 9:**
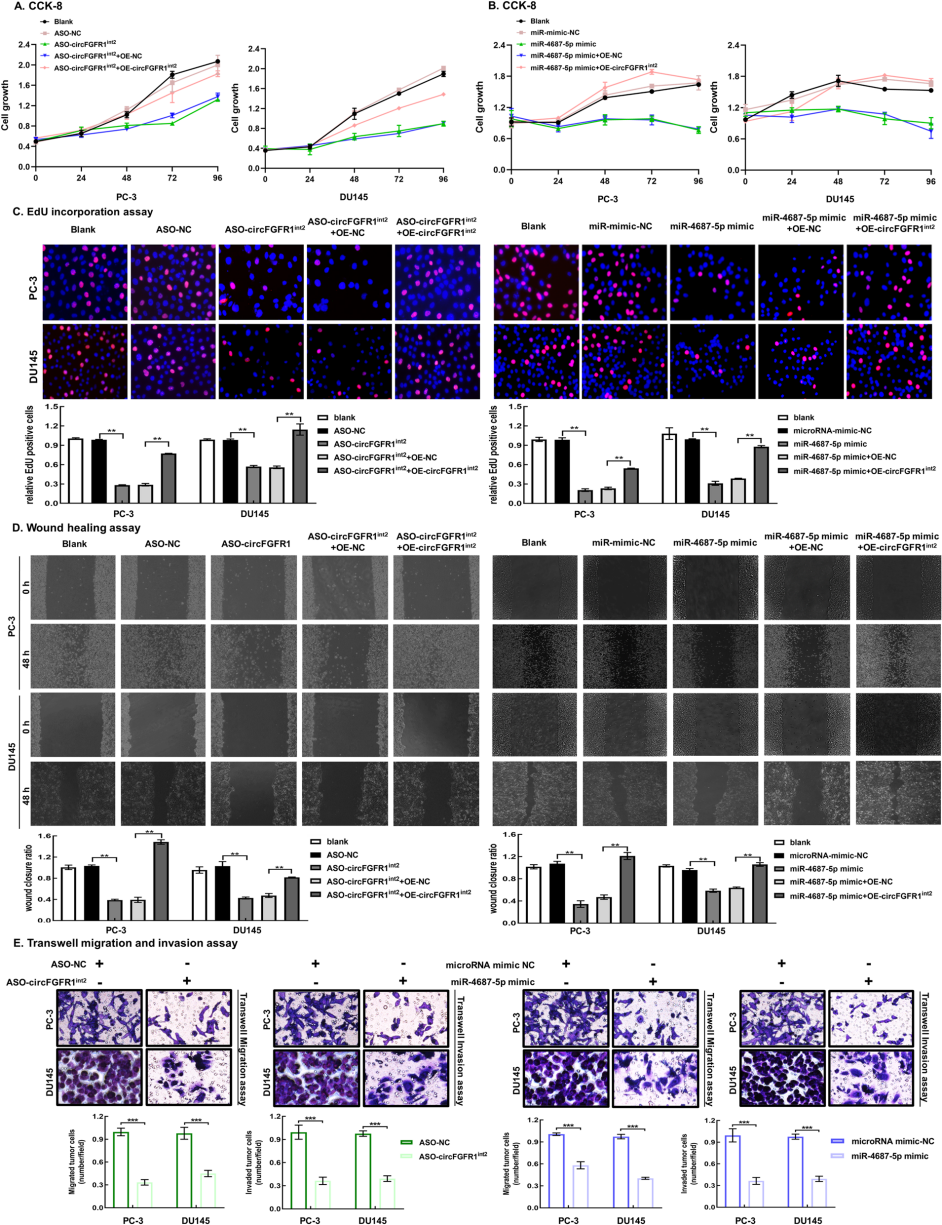
Upregulated circFGFR1^int2^ and downregulated miR-4687-5p promoted PCa cell proliferation, migration, and invasion. **A**–**C** Knockdown of circFGFR1^int2^ by ASO-circFGFR1^int2^ or artificial overexpression of miR-4687-5p by mimic significantly decreased PCa cell proliferation and DNA replication as shown by CCK-8 assays and EdU incorporation assays, which could be reversed by artificial overexpression of circFGFR1^int2^. **D** Knockdown of circFGFR1^int2^ by ASO-circFGFR1^int2^ or artificial overexpression of miR-4687-5p by mimic significantly decreased PCa cell migration and invasion as shown by wound healing, which could be reversed by artificial overexpression of circFGFR1^int2^. **E** Similar effects as shown by Transwell assays. Three microscopic fields were evaluated for each sample. Error bars for CCK-8, EdU incorporation, wound healing and Transwell assays represented mean ± standard deviation (SD) of three independent experiments. **P < 0.01, ***P < 0.001

Knockdown of circFGFR1^int2^ by ASO-circFGFR1^int2^ or artificial overexpression of miR-4687-5p mimic significantly decreased proliferation of PC-3 and DU145 cells, which could be reversed by artificial circFGFR1^int2^ overexpression (Fig. 9A and B). PCa cell DNA replication as shown by EdU incorporation assays was significantly reduced by knockdown of circFGFR1^int2^ or overexpression of miR-4687-5p mimic, which could also be reversed by artificial overexpression of circFGFR1^int2^ (Fig. 9C).

Wound healing experiments showed that ASO-circFGFR1^int2^ or miR-4687-5p mimic transfection significantly decreased PCa cell migration, which could be rescued by artificial circFGFR1^int2^ overexpression (Fig. 9D). Similarly, Transwell experiments demonstrated significant decrease of PCa cell migration and invasion by circFGFR1^int2^ knockdown or artificial miR-4687-5p overexpression (Fig. 9E).

### Overexpression of circFGFR1^int2^ was associated with PCa progression and poor survival

The positive rate of circFGFR1^int2^ as assayed by in situ hybridization in PCa was significantly higher than that in BPH (*P* < 0.001) (Table 1). Spearman rank correlation analysis revealed a significantly positive correlation between circFGFR1^int2^ expression and WHO grade group (*P* < 0.001, R = 0.683), Gleason score (*P* < 0.001, R = 0.670), and PSA level (*P* = 0.037, R = 0.331) (Table 2).

**Table 1:** Comparison of CircFGFR1^int2^ expression in PCa and BPH

	CircFGFR1^int2−high^	CircFGFR1^int2−low^
PCa	45 (72.6%)	17 (27.4%)
BPH	7 (17.5%)	33 (82.5%)
	*P* values	
PCa vs. BPH	** < 0.001**	** < 0.001**

CircFGFR1^int2^ was assessed by in situ hybridization, with integrated product of staining intensity and extent ≥ 4 defined as CircFGFR1^int2−high^, and those < 4 as CircFGFR1^int2−low^ (see “Materials and Methods” section)

*P* value was obtained by Fisher exact test, and bold types highlight *P* values < 0.05

**Table 2:** Correlation of CircFGFR1^int2^ expression with clinicopathological parameters

	CircFGFR1^int2^	Grade	PSA
WHO grade group	0.683^***^		
Gleason score	0.670^***^	0.963^***^	
PSA	0.331^*^	0.325^*^	
Age	0.031	− 0.294^*^	0.330^*^

The numbers are Spearman rank correlation coefficients, and bold types highlight coefficients for which *P* values are < 0.05

*P < 0.05, ***P < 0.001

**Fig. 10:**
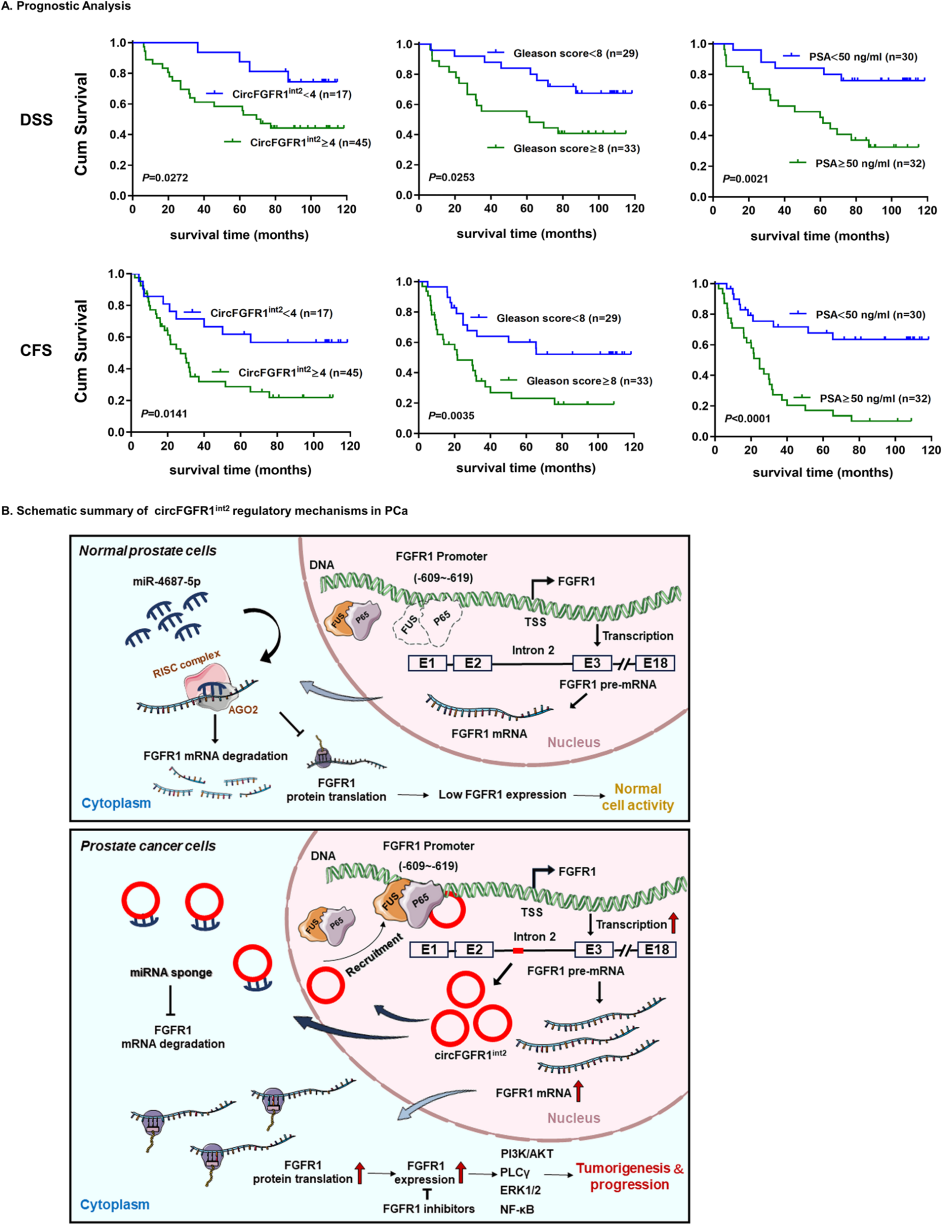
Prognostic significance of circFGFR1^int2^ overexpression in PCa and schematic summary of the major findings of the present study. **A** Survival analysis by Kaplan–Meier method with log-rank test revealed that disease-specific survival (DSS) and castration-resistance free survival (CFS) of PCa patients were significantly shorter when CircFGFR1^int2^ ≥ 4, Gleason score ≥ 8, and PSA ≥ 50 ng/ml. **B** Schematic summary of the major findings of the present study

Survival analysis by Kaplan–Meier method revealed that higher circFGFR1^int2^ expression (circFGFR1^int2−high^, ISH score of circFGFR1^int2^ ≥ 4), Gleason score ≥ 8, and PSA ≥ 50 ng/ml were significant prognostic factors for both DSS and CFS for the patient cohort of the present study (Fig. 10A). Multivariate analysis by Cox proportional hazard model showed that circFGFR1^int2−high^ was an independent unfavorable prognosticator for CFS (relative risk = 3.277, 95% confidence interval: 1.192–9.009, *P* < 0.021) (Table 3).

**Table 3:** Survival analysis by Cox proportional hazard model

	*P* value	*RR*	95% CI	
			Lower	Upper
CircFGFR1^int2^ (high vs. low)	**0.021**	**3.277**	1.192	9.009
Gleason score (≥ 8 vs. < 8)	0.685	1.180	0.531	2.620
PSA (≥ 50 ng/ml vs. < 50 ng/ml)	** < 0.001**	4.022	1.853	8.731

RR: relative risk; 95% CI: 95% confidence interval

*P* values < 0.05 were in bold

## Discussion

Deep RNA sequencing and bioinformatics analysis revealed a large number of circRNAs transcripts in various species [22, 23]. Although aberrant expression of some circRNAs have been shown to be involved in human malignancies [24–26], the functions and clinical implications of many circRNAs have yet to be elucidated [6]. In the present study, we discovered circFGFR1^int2^, a novel circRNA derived from intron 2 of *FGFR1*, was up-regulated in PCa and promoted PCa progression by facilitating FGFR1 transcription through recruiting transcription activators P65/FUS that interacted with *FGFR1* promoter. Moreover, circFGFR1^int2^ suppressed miR-4687-5p, a novel post-transcriptional inhibitor of FGFR1 mRNA. These mechanisms synergistically promoted PCa cell growth, migration, and invasion by up-regulating expression of FGFR1. Overexpression of circFGFR1^int2^ was significantly correlated with higher grade, Gleason score, and PSA level, and was a significantly unfavourable prognosticator for PCa patient survival. These findings unravelled mechanisms by which the novel circFGFR1^int2^ promoted *FGFR1* gene expression and showed its potential clinicopathological utility as a diagnostic or therapeutic target.

Overexpression of *FGFR1* has been reported in a variety of cancers, including carcinomas of lung [27], breast [28], oral cavity [29], the ovaries, urinary bladder and prostate [30], as well as mesenchymal or lymphoid malignancies such as rhabdomyosarcoma [31] and acute myeloid leukemia [32]. Overexpression of *FGFR1* promoted tumor cell growth, migration, invasion, angiogenesis, and metastasis, and was associated with poor prognosis [33, 34].

Expression of *FGFR1* was regulated at multiple levels, including genomic, transcriptional, and post-transcriptional mechanisms. Genomic abnormalities such as gene amplification [7, 8], gene rearrangement [9], and point mutation [35] were detected in multiple tumors. In a study of 4,853 solid tumors (including carcinomas of urothelium, breast, and ovary) by next-generation sequencing, FGFR abnormalities, predominantly gene amplification (66%), mutation (26%), and rearrangement (8%), were present in 3.5% of the patients and in 7.1% of the cancers [36].

Transcription activators and specific *cis* response elements (RE) of *FGFR1* have been described in humans, including TEAD and co-activator YAP (RE at − 1000) [37], E2F-1 (RE at + 4 ~  + 22 and + 25 ~  + 43, respectively)[38], and RTEF-1 (RE at − 48 to − 20)[39]. Several transcription activators and suppressors have been described in animals, such as KLF9 [40], KLF10 [41], AP-2α [42], Sp1/Sp3 [43], E2F4/p107 and E2F4/p130 [44], which were involved in chicken myoblast differentiation and proliferation. FGFR1 transcription was activated by FOXC1 [45], Sp1 [46], TRα1, TRβ1, and TRβPV [47] but supressed by Sp3 in mice [48].

In head and neck squamous cell carcinomas, *FGFR1* gene hyper-methylation was found at 18 of 42 CpG sites and hypo-methylation at 16 of 42 CpG sites [49], and *FGFR1* demethylation was associated with acquired cetuximab resistance [14].

Suppression of *FGFR1* expression by miR-133a-3p [15], miR15 and miR16 [16], which targeted the *FGFR1* 3′UTR, was disrupted in PCa. LncRNA MIR210HG interacted with octamer transcription factor 1, promoting *FGFR1* transcription and glioblastoma multiforme progression [17]. A couple of non-FGFR1-derived circRNAs have been reported to promote *FGFR1* expression, such as circ_SNX27 [18] and circRAPGEF5 [19], which functioned as miRNA sponges for miR-637 (in hepatocellular carcinoma) and miR-198 (in papillary thyroid carcinoma) respectively.

Only a few *FGFR1*-derived circRNAs have been reported, including hsa_circ_0084003 (derived from exons 5 to 19) and hsa_circ_0084007 (derived from exons 2 to 7). These are all derived from exons (ecircRNA). The hsa_circ_0084003 was upregulated in non-small cell lung cancer and was a miR-381-3p sponge to block its inhibitory effects on C–X–C motif chemokine receptor 4, thus promoting tumor cell immune evasion [50], and promoted pancreatic ductal adenocarcinoma progression by targeting miR-532-3p/PIK3CB [51]. The hsa_circ_0084007 encoded a protein circFGFR1p, which negatively regulated *FGFR1* and suppressed cell proliferation during heat-shock induced stress in HEK-293 T cells [52].

CircRNAs may also derive from exon–intron (EIciRNAs) and introns (ciRNAs) [53]. EIciRNAs and ciRNAs derived from *FGFR1* introns have not been described before. The present study was the first to report an intron-derived circRNA from *FGFR1* which we found to be overexpressed in PCa to promote tumor cell growth and invasion*.*

Intron-containing circRNAs (ciRNA and EIciRNA) tended to be retained in cell nucleus to exert enhancer-like functions on parental genes or adjacent genes [54], but may also be exported to the cytoplasm to function as sponges for miRNAs [55] or proteins [56], or to encode proteins themselves [57]. The G-rich repeats in intron could stabilize the conformation and mediate export of circC9ORF72 in co-operation with the RNA export NXF1-NXT1 pathway [57]. The almost equal nuclear and cytoplasmic distribution of circFGFR1^int2^ shown by our data indicated this novel circRNA was the first clue that it might exert diverse functions related to its subcellular localization. As our data showed, this novel intronic circRNA promoted the parental *FGFR1* gene expression by dual functions: recruitment of transactivators P65 and FUS in the nucleus, and post-transcriptional suppression of *FGFR1*-inbitory miR-4687-5p in the cytoplasm.

Various ncRNAs (including lncRNAs, snRNAs, and snoRNAs) may affect transcriptional regulation by binding to gene promoters, interacting with transcription factors, DNA demethylases/methyltransferases, histones, or other RNA binding proteins [58, 59].

CircRNAs may directly or indirectly affect the structure and function of DNA. For example, ecircRNA circSamd4 (derived from exon3 of mouse *Samd4*) interacted with transcription suppressor PURA and PURB, inhibiting their interaction with the myosin heavy chain (*MHC*) promoter, thus indirectly promoting *MHC* transcription [60]. In addition, circRNAs could directly bind to DNA coding sequences (forming a ‘circR- loop’) or promoters. By forming RNA–DNA hybrid at the cognate loci, the circMLL (9, 10) inhibited the activities of RNA poly II and proteasome, promoted DNA breakage and chromatin re-organization of the *MLL* gene [61]. Several EIciRNAs (circEIF3J and circPAIP2) bound to RNA Pol II and U1 snRNP at 0–300 region before *EIF3J* and *PAIP2* transcriptional start site to promote U1 snRNP mediated transcription in *cis* [54]. An ecircRNA (circAnks1a) in spinal dorsal horn neurons promoted nuclear import and recruitment of the transcription factor YBX1 to *VEGFB* promoter to activate *VEGFB* transcription [62].

Whether ciRNAs interacted with DNA to regulate transcription in cancers remains unclear. Only a few intron-derived circRNAs have been characterized, and their roles in cancers remain largely elusive. For example, ci-Ins2 (circInsulin) derived from *ins2* gene intron 2 was mainly found in the nucleus in murine β-cell line MIN6B1 and co-operated with the DNA-binding protein TDP-43 to regulate insulin secretion in pancreatic islets [63]. ci-Ins2 was downregulated in islets of diabetic Goto-Kakizaki (GK) rats. Silencing of ci-Ins2 supressed Na^+^/K^+^ ATPase subunits, Ca^2+^ channel-related genes, and small GTPase signaling components, leading to impaired insulin secretion [63]. The oncogenic circAGO2 (hsa_circ_0135889) derived from *AGO2* gene intron 1 bound to HuR and promoted its export to cytoplasm and binding to mRNA 3′UTR, thus suppressing AGO2-miR interaction and mRNA degradation of multiple oncogenes in gastric cancer cells [64]. The present study found the novel function of the intron-derived ciRNA circFGFR1^int2^ in regulating parental gene *FGFR1* expression by interacting with the *FGFR1* promoter and recruitment of transcription activators FUS/P65.

The multifunctional FUS protein was a DNA- and RNA-binding protein involved in diverse biological processes such as gene transcription, DNA stability, and RNA alternative splicing [65, 66], through its interaction with RNA/DNA, transcription factors (such as MITF [67], P65 [21, 68], TFIID, RNA pol II [69], and splicing factors (such as PTB and SR proteins) [70]. One study showed that circ0005276 derived from *XIAP* (X-linked inhibitor of apoptosis protein) interacted with FUS to activate *XIAP* transcription and promoted PCa progression [71].

A major function of FUS is to act as a transcriptional co-activator for specific transcription factors, such as P65 (RELA) [21, 68], a core component of NF-κB (nuclear factor kappa-B). NF-κB played key roles in many biological processes such as immune response, inflammatory reaction, cell apoptosis, cell proliferation and differentiation [72]. In PCa, upregulation and nuclear import of P65 was associated with tumor progression and was an independent predictor for biochemical recurrence [73, 74]. Although TCGA data showed that the expressions of P65 and FGFR1 in PCa tissues were positively correlated (Fig. 3G), it was unknown whether P65 regulated FGFR1.

The present study showed for the first time that P65 activated *FGFR1* transcription by binding to a consensus ‘GACGTTCCCTA’ sequence in *FGFR1* promoter (− 609 to  − 619), and the novel circFGFR1^int2^ facilitated *FGFR1* transcription by interacting with both promoter and the transcription activators, thus recruiting the transcription activators P65/FUS to the *FGFR1* promoter.

Most circRNAs were reported to be enriched in the cytoplasm. Cytoplasmic circRNAs were involved in multiple functions. A much-studied function of MRE (microRNA response element)-containing circRNA was its suppressive miR sponging function. For example, CiRS-7 (CDr1as), a typical ‘molecular sponge’ that contained over 60 miR-7 binding sites, acted as a sponge for miR-7, thus relieving its suppressive effects on EGFR, SNCA, and IRS2 [75]. Cytoplasmic circRNAs may also influence the stability, function, and subcellular localization of proteins. In addition, circRNAs containing IRES or m6A-modified sites may encode proteins of diverse functions [76].

The targets of miR-4687-5p had not been reported before. Our study identified *FGFR1* as the target of miR-4687-5p, which was previously reported to be downregulated in ALS (amyotrophic lateral sclerosis) [77], cystic echinococcosis [78], polycystic ovary syndrome [79], and breast cancer [80]. Decreased miR-4687-5p in blood was found to be of diagnostic value in sporadic ALS with an accuracy of 0.66 [81]. However, potential gene targets of miR-4687-5p in neoplastic and non-neoplastic diseases remain to be explored. Our study not only found that *FGFR1* mRNA was suppressed by miR-4687-5p which targeted both the 3′UTR and the CDS of *FGFR1* pre-mRNA, but also discovered that circFGFR1^int2^ sponged and inhibited miR-4687-5p. These data thus provided double-level mechanisms by which circFGFR1^int2^ enhanced *FGFR1* expression, one by participating in transcription activation, and the other by inhibiting suppressive miR.

FGFR1 might promote PCa progression through activation of oncogenic pathways and increase of resistance to anti-tumor drugs. FGFR1 had been reported to activate ERK1/2, PI3K/AKT, PLCγ, NF-κB, and Wnt/β-catenin signalling in PCa [6, 82, 83]. Inhibition of FGFR1 suppressed PCa-bone cell interaction and increased antitumor effects of dovitinib (TK1258), a receptor tyrosine kinase inhibitor that potently inhibited FGFR [84]. As drugs and preparations targeting FGFs/FGFRs signaling showed potential effects against PCa progression [84, 85], future studies evaluating potential diagnostic and therapeutic uses of circFGFR1^int2^ may also be envisaged. Besides, potential diagnostic use of circRNAs in body fluids [86] will be of particular interest.

The biogenesis of circFGFR1 will be an intriguing aspect to be explored in the future. As FUS has been implicated in alternative splicing, RNA metabolism [65, 66] and circRNA biogenesis [87], it would be interesting to speculate whether FUS participated in circFGFR1 formation or metabolism by interacting with the *FGFR1* pre-mRNA. Hypothetically, other factors, including RNA-binding proteins (RBPs)/splicing factors [88], m6A or other forms of pre-mRNA mRNA modifications [89], and other *cis* elements that facilitated circRNA back splicing, may also be involved in promoting circFGFR1 biogenesis.

## Conclusions

The present study discovered a novel circRNA, circFGFR1^int2^, derived from intron 2 of *FGFR1*, which promoted *FGFR1* expression and PCa progression. Our data unravelled that circFGFR1^int2^ promoted *FGFR1* expression at both the transcription and the post-transcription levels: by recruiting the transcription activators P65 and FUS which transactivated *FGFR1* transcription, and by supressing miR-4687-5p, which inhibited *FGFR1* translation. These findings together showed novel mechanisms of *FGFR1* deregulation in PCa and may find diagnostic and therapeutic applications in the future.

### Supplementary Information

**Additional file 1: Figure S1. A** Circular RNAs derived from FGFR1 recorded in CircBase (http://www.circbase.org/). **B** Interference efficiency of circFGFR1^int2^. **Figure S2. **Bioinformatics analyses by ORFfinder, IRESbase, and SRAMP databases. Analyses revealed several ORFs (**A**) in the circFGFR1^int2^ sequence, but no IRES (internal ribosome entry sites) (**B**) or m6A modification sites (**C**). **Figure S3.** Sanger sequencing of the wild type and mutated sites. **Table S1.** Sequences of siRNAs, ASOs, and RNA probes. **Table S2.** PCR primers. **Table S3.** Primers used in RNA Dot blot experiment. **Table S4.** Primers used in Dual-luciferase reporter assay.

## Data Availability

Data reported in the study shall be available upon reasonable request.

## Abbreviations

ALS: Amyotrophic lateral sclerosis
AR: Androgen receptor
ASO: Antisense oligonucleotides
CFS: CRPC-free survival
ChIP: Chromatin immunoprecipitation
ChIRP: Chromatin isolation by RNA purification
Co-IP: Co-immunoprecipitation
CRPC: Castration resistant prostate cancer
DSS: Disease specific survival
EZH2: Enhancer of zeste homolog 2
FGFR1: Fibroblast growth factor receptor 1
FUS: Fused in sarcoma
IHC: Immunohistochemistry
ISH: In situ hybridization
MHC: Myosin heavy chain
MRE: microRNA response element
NF-κB: Nuclear factor kappa-B
PCa: Prostate adenocarcinoma
RIP: RNA immunoprecipitation
RE: Response element
RBPs: RNA-binding proteins
XIAP: X-linked inhibitor of apoptosis protein
