# Supplementary material for: A novel intronic circular RNA circFGFR1int2 up-regulates FGFR1 by recruiting transcriptional activators P65/FUS and suppressing miR-4687-5p to promote prostate cancer progression
Source: J Transl Med. 2023 Nov 22;21:840. doi: 10.1186/s12967-023-04718-y (PMC10664560; doi:10.1186/s12967-023-04718-y)
Supplement: Supplementary file 1 — Additional file 1: Figure S1. A Circular RNAs derived from FGFR1 recorded in CircBase (http://www.circbase.org/). B Interference efficiency of circFGFR1int2. Figure S2. Bioinformatics analyses by ORFfinder, IRESbase, and SRAMP databases. Analyses revealed several ORFs (A) in the circFGFR1int2 sequence, but no IRES (internal ribosome entry sites) (B) or m6A modification sites (C). Figure S3. Sanger sequencing of the wild type and mutated sites. Table S1. Sequences of siRNAs, ASOs, and RNA probes. Table S2. PCR primers. Table S3. Primers used in RNA Dot blot experiment. Table S4. Primers used in Dual-luciferase reporter assay. [file 12967_2023_4718_MOESM1_ESM.docx]

**Supplementary Figures**

**
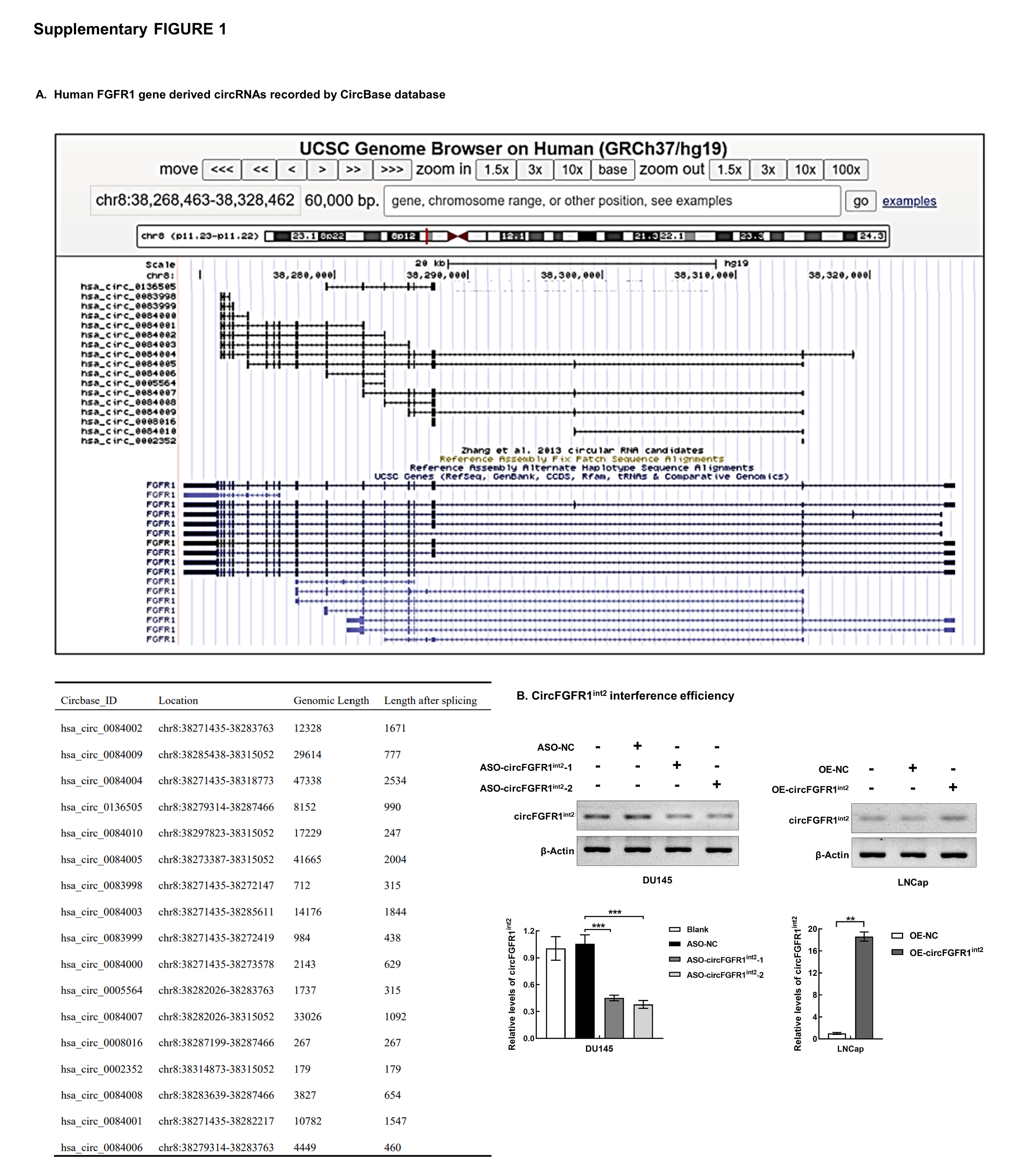
**

**Supplementary FIGURE 1 (A)** Circular RNAs derived from FGFR1 recorded in CircBase (<http://www.circbase.org/>). **(B)** Interference efficiency of circFGFR1^int2^.

**
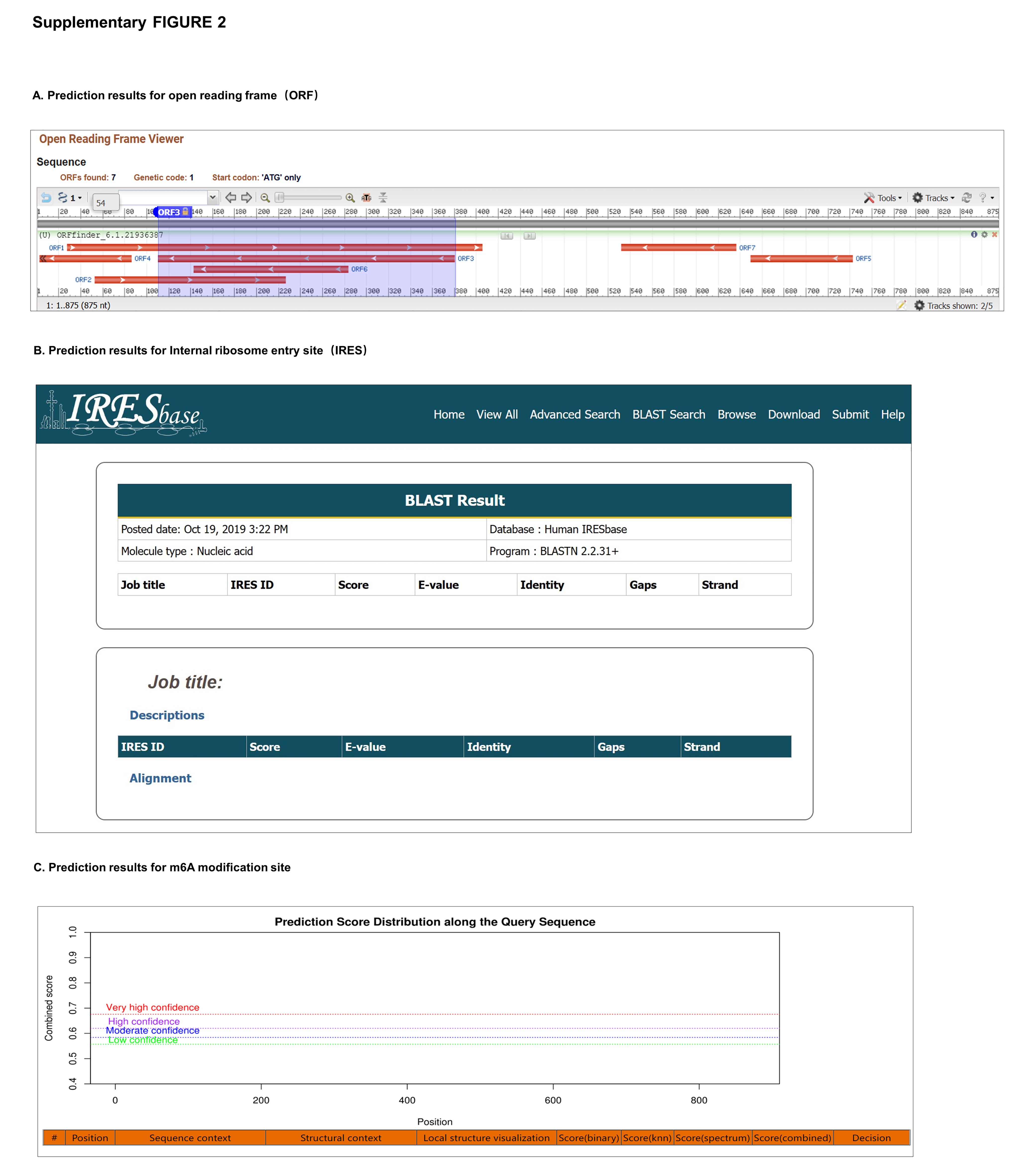
**

**Supplementary FIGURE 2 Bioinformatics analyses by ORFfinder, IRESbase, and SRAMP databases.** Analyses revealed several ORFs **(A)** in the circFGFR1^int2^ sequence, but no IRES (internal ribosome entry sites) **(B)** or m6A modification sites **(C)**.

**
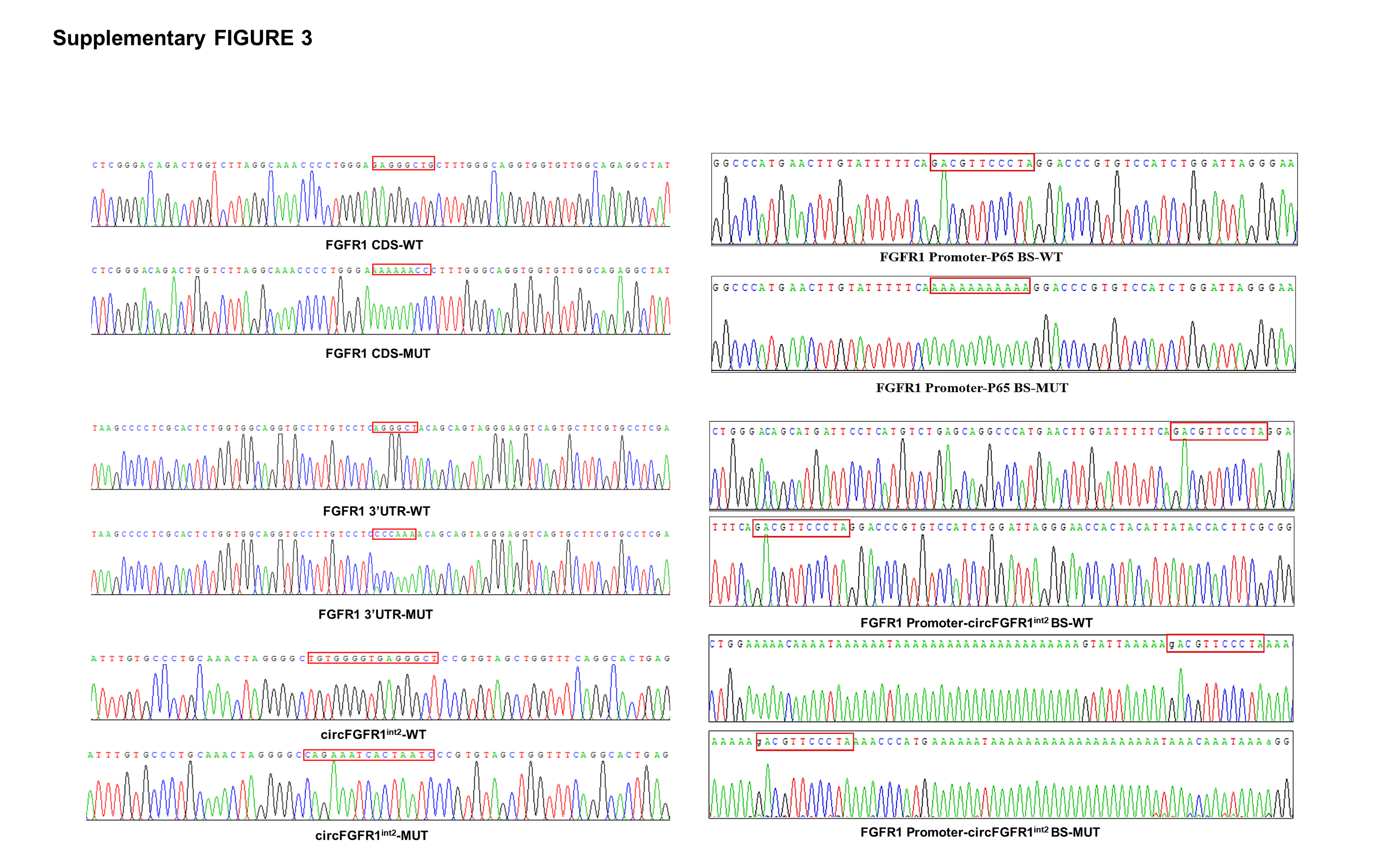
**

**Supplementary FIGURE 3 Sanger sequencing of the wild type and mutated sites.**

**Supplementary Tables**

**Supplementary Table 1. Sequences of siRNAs, ASOs, and RNA probes**

| **Name** | **Sequence (5’-3’)** |  |
| --- | --- | --- |
| si-FUS | GATCAATCCTCCATGAGTA |  |
| si-P65 | GCATTAACTTCTCTGGAAA |  |
| ASO-circFGFR1^int2^-1 | TCACCCTGTAGACACCACCT |  |
| ASO-circFGFR1^int2^-2 | AGGCACTGAGAAAGATCCAC |  |
| circFGFR1^int2^ probe | TTCTGCCTGGCCAGGAGGCTCAGCCACGCCACAGCAT |  |
| circFGFR1^int2^-control probe | ATGCTGTGGCGTGGCTGAGCCTCCTGGCCAGGCAGAA | |
| circFGFR1^int2^-random probe | CCGCGGCTACACGATTCCCCCGGGATCCGTAGAGTAC |  |

**Supplementary Table 2. PCR primers**

| **Name** | **Sequence (5’-3’)** | **Product**  **Length (bp)** | |
| --- | --- | --- | --- |
| circFGFR1^int2^ divergent primers | FP: CTTGAAACTCCACGCCCTCT  RP: AGACACAAGCCATCACTCACTG | | 90 |
| circFGFR1^int2^ convergent primers | FP: CAGTGAGTGATGGCTTGTGTCT  RP: AGAGGGCGTGGAGTTTCAAG | | 827 |
| FGFR1 | FP: CACCCGAGGCATTATTTGAC  RP: AAGTTCCTCCACAGGCACAC | | 125 |
| Globin | FP: GATCTGTCCACTCCTGATGCTG  RP: ATCAAGCGTCCCATAGACTCAC | | 196 |
| GAPDH | FP: GGAGCGAGATCCCTCCAAAAT  RP: GGCTGTTGTCATACTTCTCATGG | | 197 |
| β-Actin | FP: CTGGCACCACACCTTCTACAATG  RP: CCTCGTAGATGGGCACAGTGTG | | 248 |
| SNORA41 | FP: ACTGGTCTGCAGCTGTTCTTA | | 120 |
|  | RP: GTGTCTGTCACACATATATACCCAC | |  |
| circFGFR1^int2^_full length | FP: CTCCTGGCCAGGCAGAAGCAGTGA  RP: GCTCAGCCACGCCACAGCATGT | | 875 |
| P65-BS genomic DNA | FP: GAGCAGGCCCATGAACTTGTATTTT | | 100 |
|  | RP: CTGAGTCTTCCCGCGAAGTGGTATA | |  |
| miR-4687-5p RT primer | GTCGTATCCAGTGCAGGGTCCGAGGTATTCGCACTGGATACGACTTTGGG | |  |
| miR-4687-5p PCR primers | FP: TTATCTCAGCCCTCCTCCCGC  RP: GTGCAGGGTCCGAGGT | | 62 |
| U6 | FP: TGGAACGATACAGAGAAGATTAGCA | | 66 |
|  | RP: AACGCTTCACGAATTTGCGT | |  |

**Supplementary Table 3. Primers used in RNA Dot blot experiment**

| **Name** | | **Sequence (5’-3’)** |
| --- | --- | --- |
| circFGFR1^int2^-WT | FP: taatacgactcactatagggCCTCAGAGTTCCCTCCCTTCTCCT  RP: GCTCAGCCACGCCACAGCA | |
| circFGFR1^int2^-MUT | FP1: taatacgactcactatagggCCTCAGAGTTCCCTCCCTTCTCCTGTTTCT  RP1: GATTAGTGATTTCTGGCCCCTAGTTTGCAGGG | |
|  | FP2: CAGAAATCACTAATCCCGTGTAGCTGGTTTCA | |
|  | RP2: GCTCAGCCACGCCACAGCAT | |
| FGFR1 3’UTR-WT | FP: taatacgactcactatagggAGCCAATGAACAGGCATGCA  RP: TACCAGGCATTTGGTCAGCAA | |
| FGFR1 3’UTR-MUT | FP1: taatacgactcactatagggAGCCAATGAACAGGCATGCAAG | |
|  | RP1: ACTGCTGTTTTGGGGAGGACAA | |
|  | FP2: TTGTCCTCCCCAAAACAGCAGT | |
|  | RP2: TACCAGGCATTTGGTCAGCAAAG | |
| FGFR1 CDS-WT | FP: taatacgactcactatagggTGGGACTCCCATGCTAGC | |
|  | RP: CATCATCTCCATTTCTGAGATCAGG | |
| FGFR1 CDS-MUT | FP1: taatacgactcactatagggTGGGACTCCCATGCTAGCAGG  RP1: GCCCAAAGGGTTTTTTTCCCAGGG | |
|  | FP2: CCCTGGGAAAAAAACCCTTTGGGC | |
|  | RP2: CATCATCTCCATTTCTGAGATCAGGTCTGA | |

**Supplementary Table 4. Primers used in Dual-luciferase reporter assay**

| **Name** | **Sequence (5’-3’)** |
| --- | --- |
| pGL3-FGFR1 promoter | FP: ggtaccCGGGGTTGAGGTGGGTGATGGTA |
|  | RP: aagcttGAGCACGTCCCAGAGGGCGC |
| pGL3-FGFR1 promoter (0~-691) | FP: ggtaccAAGGCTCTTGGCTCCTTCCTGG |
|  | RP: aagcttGAGCACGTCCCAGAGGGCG |
| pGL3-FGFR1 promoter (0~-691)-P65 MUT | FP1: ggtaccAAGGCTCTTGGCTCCTTCCTG |
|  | RP1: CCTTTTTTTTTTTTGAAAAATACAAGTTCATGGGC |
|  | FP2: CAAAAAAAAAAAAGGACCCGTGTCCATC |
|  | RP2: aagcttGAGCACGTCCCAGAGGGC |
| pGL3-FGFR1 promoter (0~-500) | FP: ggtaccGTTCTGAGTGGGCTGTGA |
|  | RP: aagcttGAGCACGTCCCAGAGG |
| pGL3-FGFR1 3’UTR-WT | FP: tctagaAGCCAATGAACAGGCATGCA  RP: tctagaTACCAGGCATTTGGTCAGCAA |
| pGL3-FGFR1 3’UTR-MUT | FP1: tctagaAGCCAATGAACAGGCATGCAAG |
|  | RP1: ACTGCTGTTTTGGGGAGGACAA |
|  | FP2: TTGTCCTCCCCAAAACAGCAGT |
|  | RP2: tctagaTACCAGGCATTTGGTCAGCAAAG |
| pGL3-CircFGFR1-WT | FP: tctagaCCTCAGAGTTCCCTCCCTTCTCCT  RP: tctagaGCTCAGCCACGCCACAGCA |
| pGL3-CircFGFR1-MUT | FP1: tctagaCCTCAGAGTTCCCTCCCTTCTCCTGTTTCT |
|  | RP1: GATTAGTGATTTCTGGCCCCTAGTTTGCAGGG |
|  | FP2: CAGAAATCACTAATCCCGTGTAGCTGGTTTCA |
|  | RP2: tctagaGCTCAGCCACGCCACAGCAT |
| pMIR-FGFR1 CDS-WT | FP: ctcgagGGGACTCCCATGCTAGC  RP: ctcgagCATCATCTCCATTTCTGAGATCAGG |
| pMIR-FGFR1 CDS-MUT | FP1: ctcgagGGGACTCCCATGCTAGCAGG  RP1: GCCCAAAGGGTTTTTTTCCCAGGG |
|  | FP2: CCCTGGGAAAAAAACCCTTTGGGC |
|  | RP2: ctcgagCATCATCTCCATTTCTGAGATCAGGTCTGA |
